# Supplementary material for: Nuclear and cytoplasmic huntingtin inclusions exhibit distinct biochemical composition, interactome and ultrastructural properties
Source: Nat Commun. 2021 Nov 12;12:6579. doi: 10.1038/s41467-021-26684-z (PMC8589980; doi:10.1038/s41467-021-26684-z)
Supplement: Supplementary file 1 — Supplementary Information [file 41467_2021_26684_MOESM1_ESM.pdf]

Supplementary Information for:

Nuclear and cytoplasmic huntingtin inclusions exhibit distinct biochemical composition, interactome and ultrastructural properties

Nathan Riguet[1], Anne-Laure Mahul-Mellier[1], Niran Maharjan[1], Johannes Burtscher[1], Marie Croisier[2], Graham Knott[2], Janna Hastings[1,3], Alice Patin[1], Veronika Reiterer[4], Hesso Farhan[4], Sergey Nasarov[1] and Hilal A. Lashuel[1]*

*Correspondence: hilal.lashuel@epfl.ch

Affiliations:

[1]Laboratory of Molecular and Chemical Biology of Neurodegeneration, Brain Mind Institute, Ecole Polytechnique Fédérale de Lausanne (EPFL), 1015 Lausanne, Switzerland.

[2]BIO EM facility (BIOEM), EPFL, 1015 Lausanne, Switzerland.

[3]Bioinformatics Competence Centre (BICC), EPFL, 1015 Lausanne, Switzerland.

[4]Institute of Pathophysiology, Medical University of Innsbruck, Innsbruck, Austria.

This document file includes:

Supplementary Figures S1 to S32

Legends for Supplementary Figures S1 to S32

Supplementary Figures

a

Primary Antibody	Reference	Company	Clone	RRID	Host	ICC Dilution	WB Dilution	Epitope
Anti-Htt	2B7	CHDI	2b7	-	Mouse monoclonal	1/500	-	Nt17 domain
Anti-Htt	Ab109115	Abcam	EPR5526	AB_10863082	Rabbit polyclonal	1/500	-	Nt17 domain
Anti-Htt	MW1	CHDI	MW1	AB_528290	Mouse	1/500	-	PolyQ
Anti-Htt	MAB5492	Millipore	2B4	AB_11213848	Mouse monoclonal	1/500	1/5000	50–64
Anti-Htt	4C9	CHDI	4C9	-	Mouse	1/500	-	PRD domain
Anti-Htt	N18 (sc-8767)	Santa-Cruz	3E10	AB_2123254	goat polyclonal	1/500	-	aa 50-100
Anti-Htt	MW8	CHDI	MW8	AB_528297	Mouse monoclonal	1/500	-	C-ter (mHtt)
Anti-Htt	S830	Bates lab	-	-	Sheep	1/500	-	mHttex1
Anti-Htt	Eurogentec (414- 4D3G9A12)	Lashuel lab	-	-	Mouse monoclonal	1/500	-	Httex1
Anti-Beta-actin	ab6276	Abcam	AC-15	AB_2223210	Mouse	-	1/5000	DDDIAALVIDNGSGK
Anti-BIP/Grp78	ab21685	Abcam	-	AB_2119834	Rabbit	1/500	-	-
Anti-Tom20	sc-17764	Santa-Cruz	F-10	AB_628381	Mouse	1/500	-	Raised against amino acids 1-145
Anti-p62	H00008878	Abnova	2C11	AB_437085	Mouse	1/500	-	Raised against a full length recombinant SQSTM1
Anti-Vimentin	ab92547	Abcam	EPR3776	AB_10562134	Rabbit	1/500	-	Synthetic peptide within Human Vimentin aa 400 to the C-terminus
Anti-HDAC6	ab1440	Abcam	-	AB_2232905	Rabbit	1/500	-	Synthetic peptide (Mouse) - N terminal
Anti-Sec13	MAB9055	R&D Systems	1280A	-	Rabbit monoclonal	1/500	-	
Anti-VDAC1	ab14734	Abcam	20B12AF2	AB_443084	Mouse	-	1/5000	Recombinant full length protein corresponding to Human VDAC1/ Porin.
Anti-MAP2	ab92434	Abcam	-	AB_92434	Chicken	1/1500	-	Recombinant MAP2 protein
Anti-NeuN	ab177487	Abcam	EPR12763	AB_2532109	Rabbit	1/500		Synthetic peptide within Human NeuN aa 1-100

b

Secondary Antibody	Reference	Company	RRID	ICC Dilution	WB Dilution
Donkey anti-rabbit Alexa Fluor 647	A31573	Invitrogen	AB_2536183	1/800	
Donkey anti-mouse Alexa Fluor 647	A31571	Invitrogen	AB_162542	1/800	
Goat anti-chicken Alexa Fluor 568	A11041	Invitrogen	AB_2534098	1/500	
Goat anti-mouse Alexa Fluor 680	A21058	Invitrogen	AB_2535724		1/5000
Goat anti-rabbit Alexa Fluor 680	A21109	Invitrogen	AB_2535758		1/5000
Goat anti-mouse Alexa Fluor 800	926-32210	Li-Cor	AB_621842		1/5000
Goat anti-rabbit Alexa Fluor 800	926-32211	Li-Cor	AB_621843		1/5000

Supplementary Figure 1. **List of the antibodies used in this study.**

Primary (**a**) and secondary (**b**) antibodies used in this study.

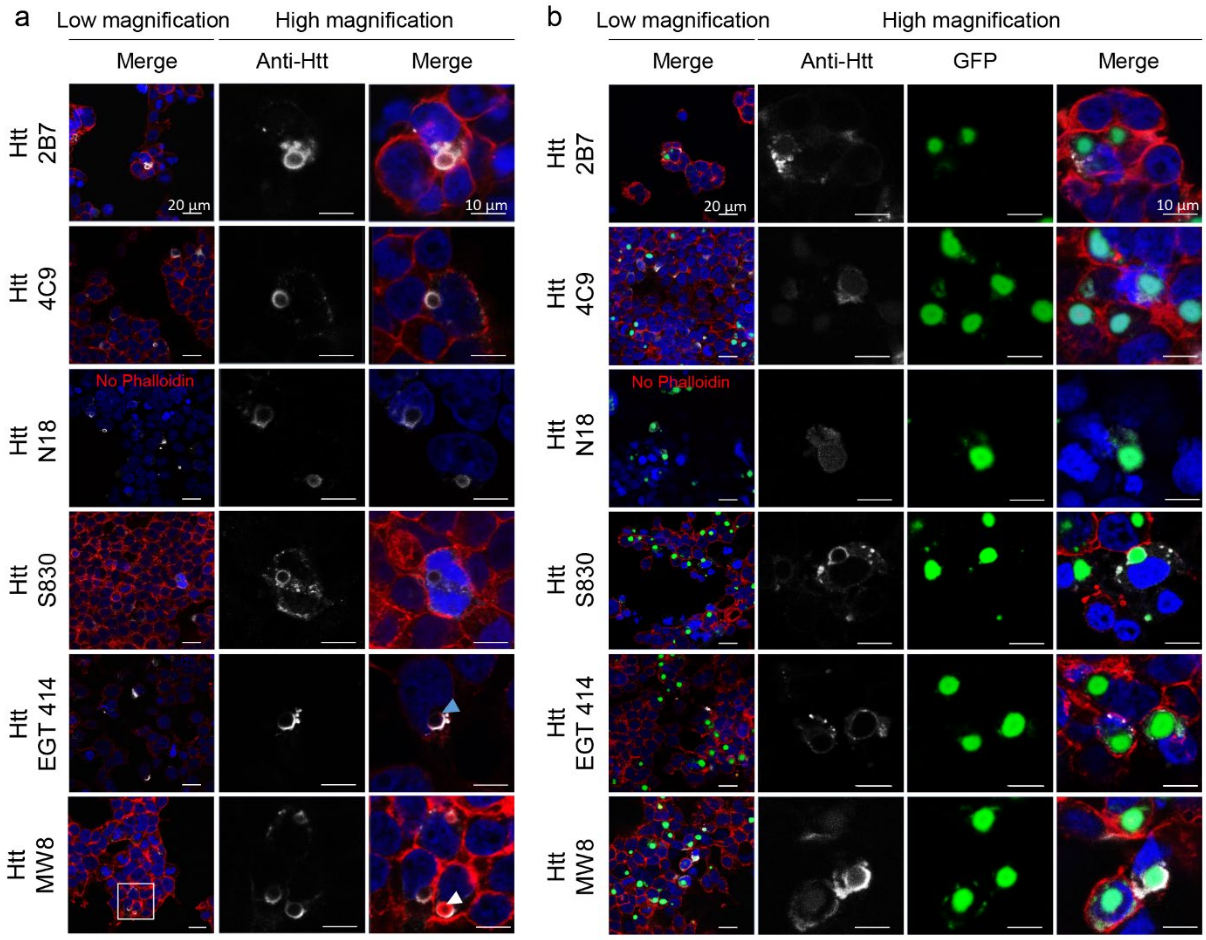

Supplementary Figure 2. **Characterization of Httex1 72Q and Httex1 72Q-GFP inclusions by Immunocytochemistry with a panel of Httex1 antibodies revealed a ring-like detection.**

a ICC of HEK cells transfected with Httex1 72Q for 48 h. **b** ICC of HEK cells transfected with Httex1 72Q-GFP for 48 h. (**a-b**) Httex1 72Q and Httex1 72Q-GFP inclusions were detected as a ring-like structure with all the Htt antibodies tested (grey) and as puncta with the GFP channel (green). Blue arrows indicate F-actin (red) colocalizing with the ring-like structure of some Httex1 72Q inclusion. DAPI was used to counterstain the nucleus. Scale bars = 20 µm (left-hand panels) and 10 µm (middle and right-hand panels).

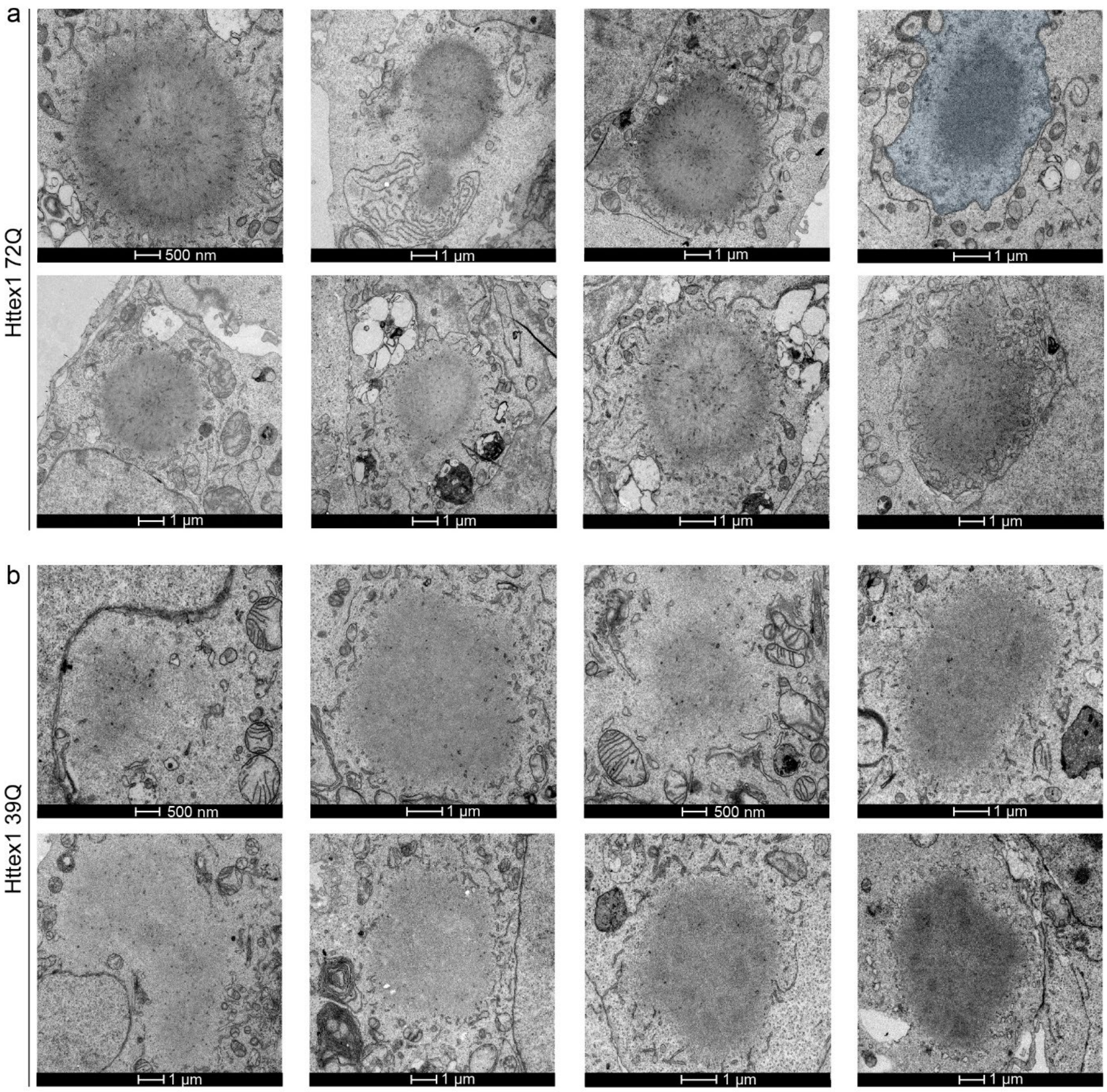

Supplementary Figure 3. **Ultrastructural characterization of Httex1 72Q and Httex1 39Q inclusions. a** 8 representative electron micrographs of Httex1 72Q inclusions in HEK cells 48 h post-transfection. **b** 8 representative electron micrographs of Httex1 39 inclusions in HEK cells 48 h post-transfection. The nucleus was highlighted in blue. Scale bars = 1 µm or 500 nm as indicated below the micrographs.

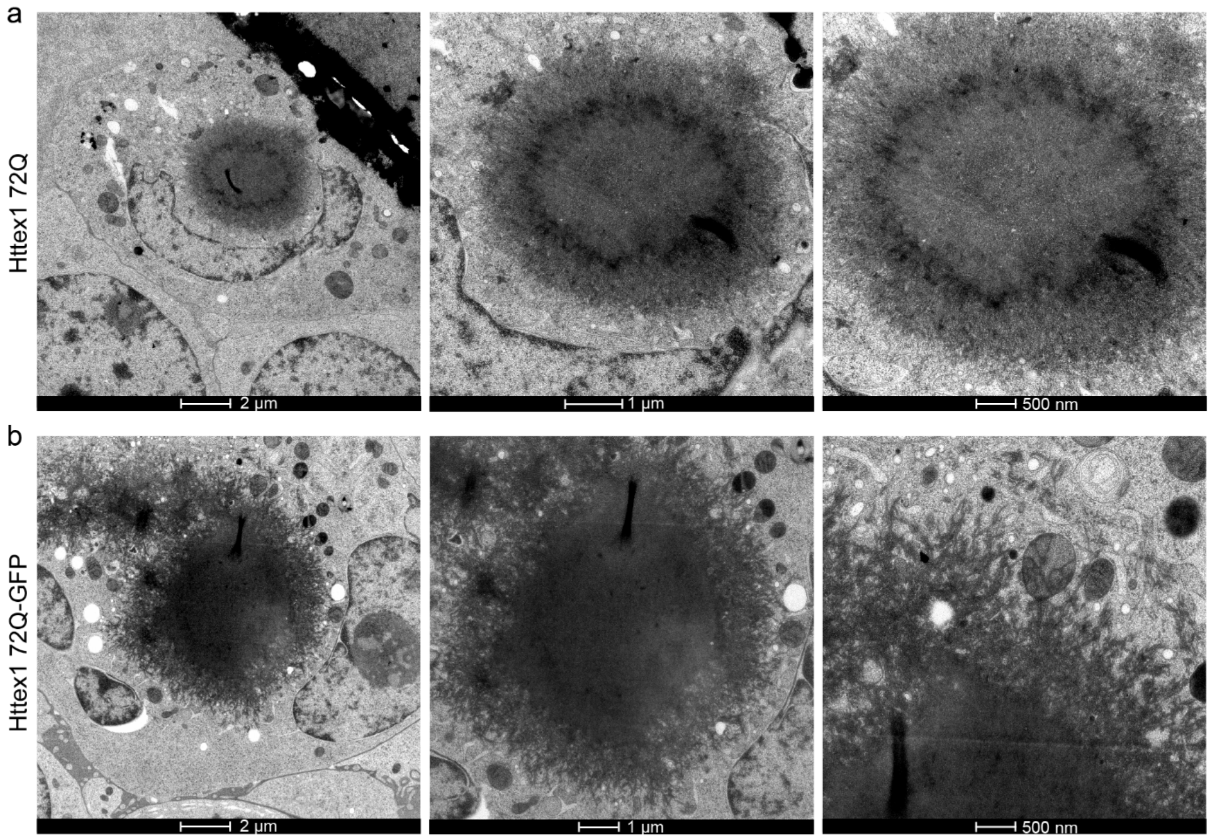

Supplementary Figure 4. **Electron microscopy analysis of Httex1 72Q and Httex1 72Q-GFP cellular inclusions post-High-Pressure Freezing demonstrates a distinct fibrillar organization**. HEK cells were fixed by HPF and freeze substituted for EM imaging 48 h after Httex1 transfection. **a** Electron micrographs of Httex1 72Q inclusion show radiating stacked fibrils. **b** Electron micrographs of Httex1 72Q-GFP inclusion reveal thick radiating fibrils at the periphery. Scale bars = 2 µm, 1 µm, or 500 nm as indicated below the micrographs.

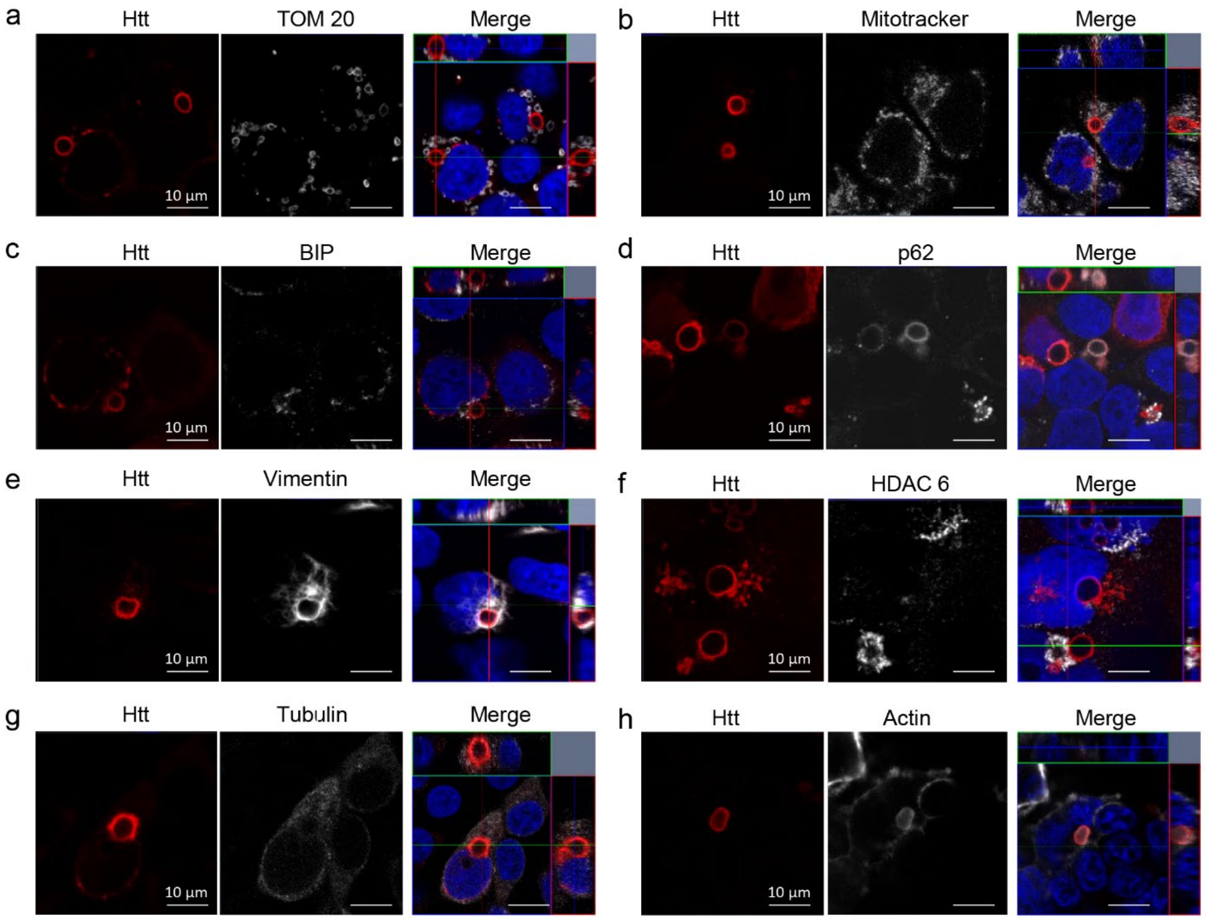

Supplementary Figure 5. **The formation of the Httex1 72Q cellular inclusions is accompanied by the accumulation of organelles at their periphery**. Httex1 72Q inclusions formed in HEK cells 48 h post-transfection were stained by Htt antibody (MAB5492, grey) in combination with organelle markers Tom20 and Mitotracker (mitochondria) (**a-b**), BIP (ER) (**c**), p62 (autophagosomes) (**d**), Vimentin (**e**) and HDAC6 (aggresome) (**f**), tubulin (**g**) and actin (cytoskeleton) (**h**). The nucleus was counterstained with DAPI (blue). Scale bars = 10 µm.

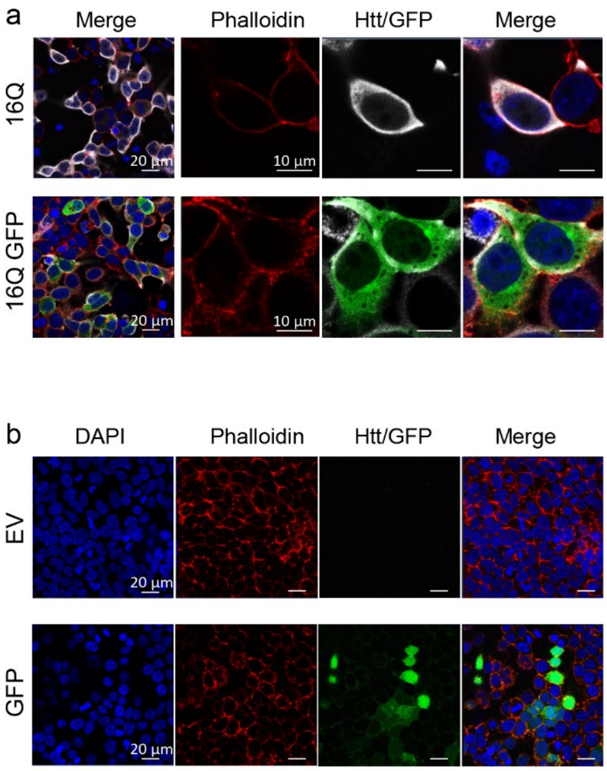

Supplementary Figure 6. **Immunocytochemistry of HEK cells expressing Httex1 16Q (+/-GFP) and EV/GFP controls does not show any aggregate formation. a** Representative confocal images of Httex1 16Q and Httex1 16Q-GFP do not display any aggregates 48 h after transfection. Scale bars = 20 µm (left-hand panels) and 10 µm (middle right-hand panels). **b** Representative confocal images of empty vector (EV) and GFP do not display any aggregates or Htt staining 48 h after transfection. Scale bars = 20 µm.

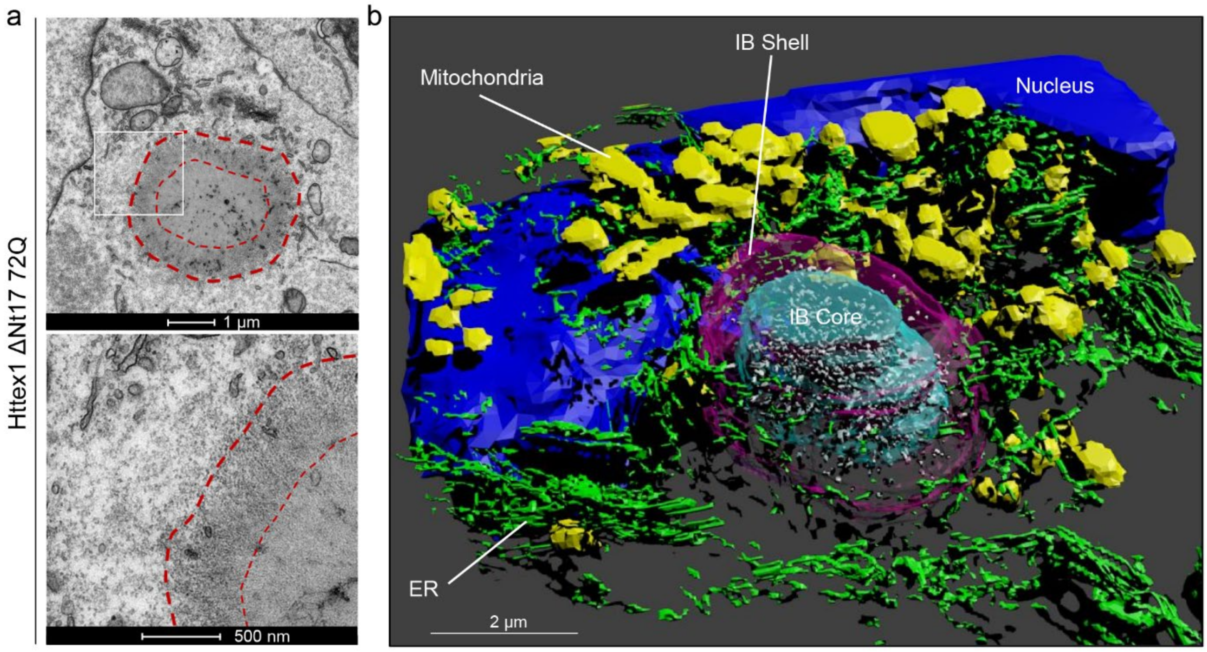

Supplementary Figure 7. **The Nt17 domain does not influence the structural architecture of the Httex1 inclusions. a** Representative electron micrograph of Httex1 ΔNt17 72Q inclusion formed 48 h after transfection in HEK. The white square indicates the higher magnification shown in the lower panel. Dashed lines delimit the inclusion and the core of the inclusion. Scale bar = 1 µm (top panel) and 500 nm (bottom panel). **b** 3D model of the Httex1 ΔNt17 72Q inclusion. The Httex1 inclusion body (IB) shell is represented in purple, the core in cyan, the ER membranes in green, the intra-inclusion membranous structures in white, the nucleus in blue, and the mitochondria in yellow. Scale bar = 2 µm.

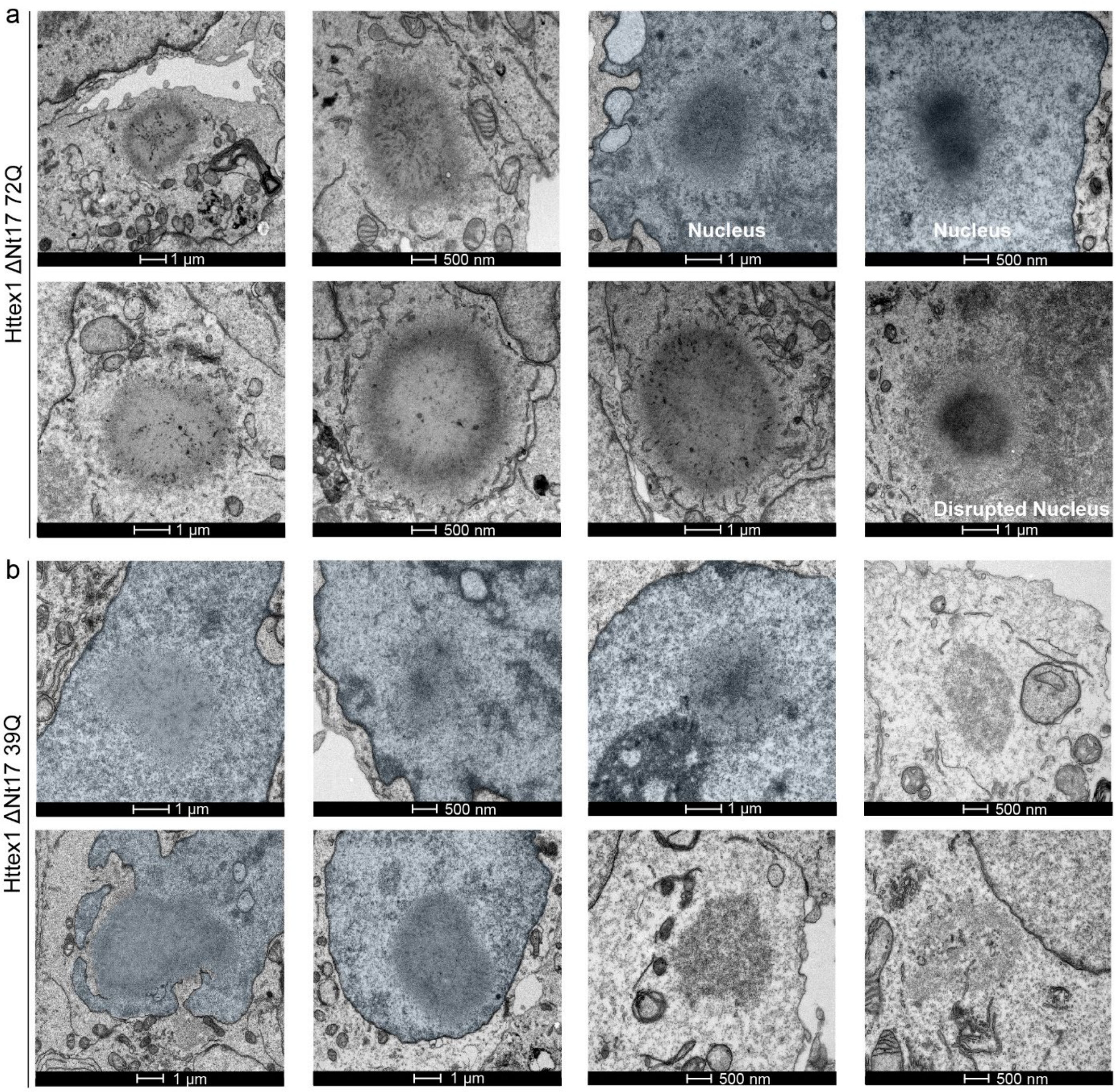

Supplementary Figure 8. **Ultrastructural characterization of Httex1 ΔNt17 72Q and Httex1 ΔNt17 39Q inclusions. a** 8 representative electron micrographs of Httex1 ΔNt17 72Q inclusions in HEK cells 48 h post-transfection. **b** 8 representative electron micrographs of Httex1 ΔNt17 39 inclusions in HEK cells 48 h post-transfection. The nucleus is highlighted in blue. Scale bars = 1 µm or 500 nm as indicated below the micrographs.

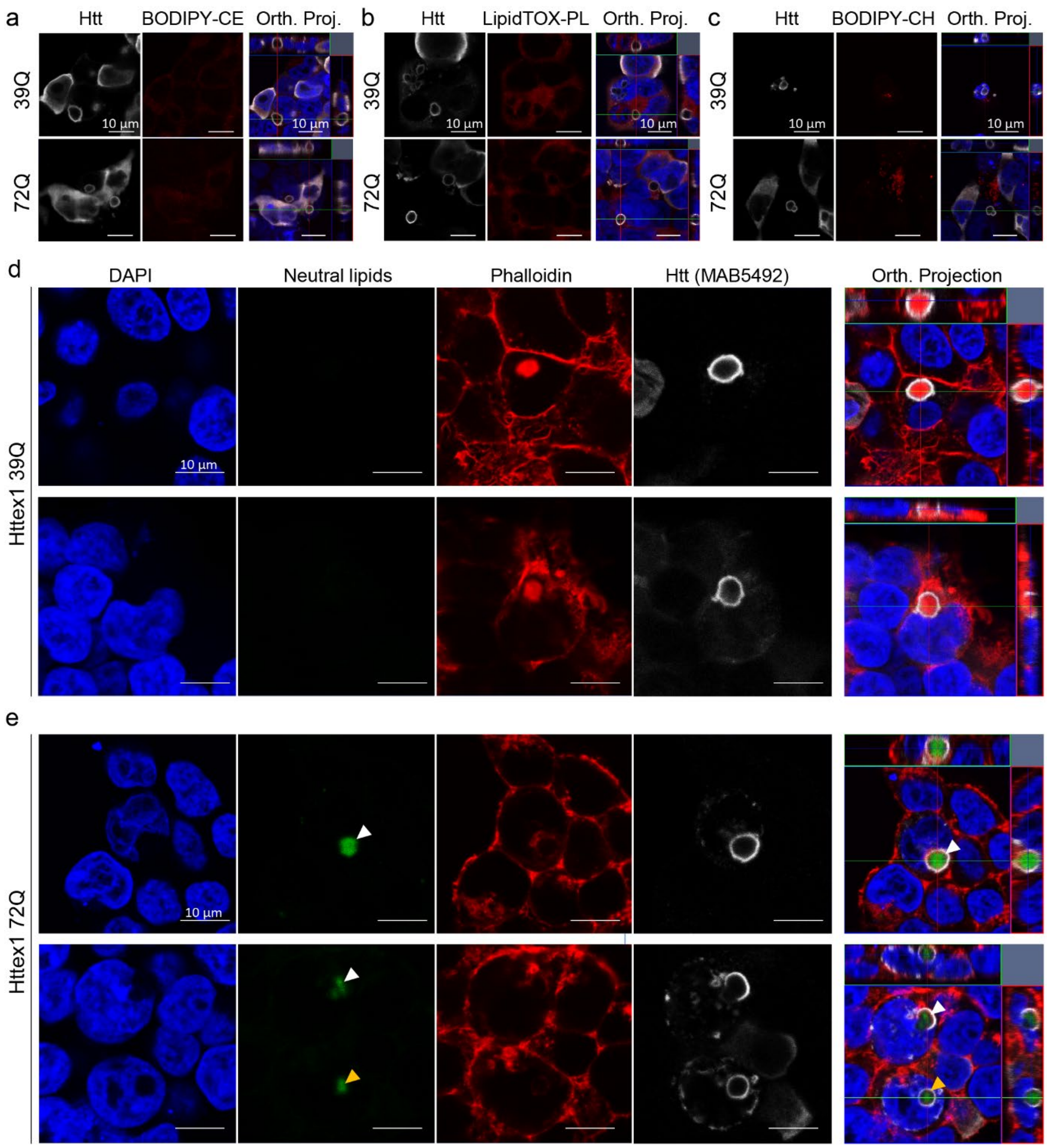

Supplementary Figure 9. **Neutral lipid enrichment of Httex1 cellular inclusions is dependent on the polyQ length.** (**a-c**) Representative confocal images of Httex1 39Q and Httex1 72Q inclusions 48 h post-transfection stained by Htt antibody (MAB5492, grey) and different lipid dyes (red). **a** The Ceramide BODIPY probe (BODIPY-CE) does not show any colocalization of inclusions with Ceramide. **b** The LipidTOX™ Red phospholipid stain (LipidTOX-PL) does not show any colocalization of inclusions with phospholipids. **c** The cholesteryl ester BODIPY probe (BODIPY-CH) does not show any colocalization of inclusions with cholesteryl ester. Scale bars = 10 µm. Representative confocal images of Httex1 39Q (**d**) and Httex1 72Q (**e**) inclusions formed 48 h after transfection in HEK cells. Inclusions were stained by the Htt antibody (MAB5492, grey) in combination with a marker of the neutral lipids (non-Polar BODIPY probe, green). The nucleus was counterstained with DAPI (blue), and phalloidin (red) was used to stain the actin F. White arrowheads indicate neutral lipid enrichment only for Httex1 72Q inclusions. Orthogonal projections (Orth. Projection) were generated from a Z-stack through the selected cells. Scale bars = 10 µm.

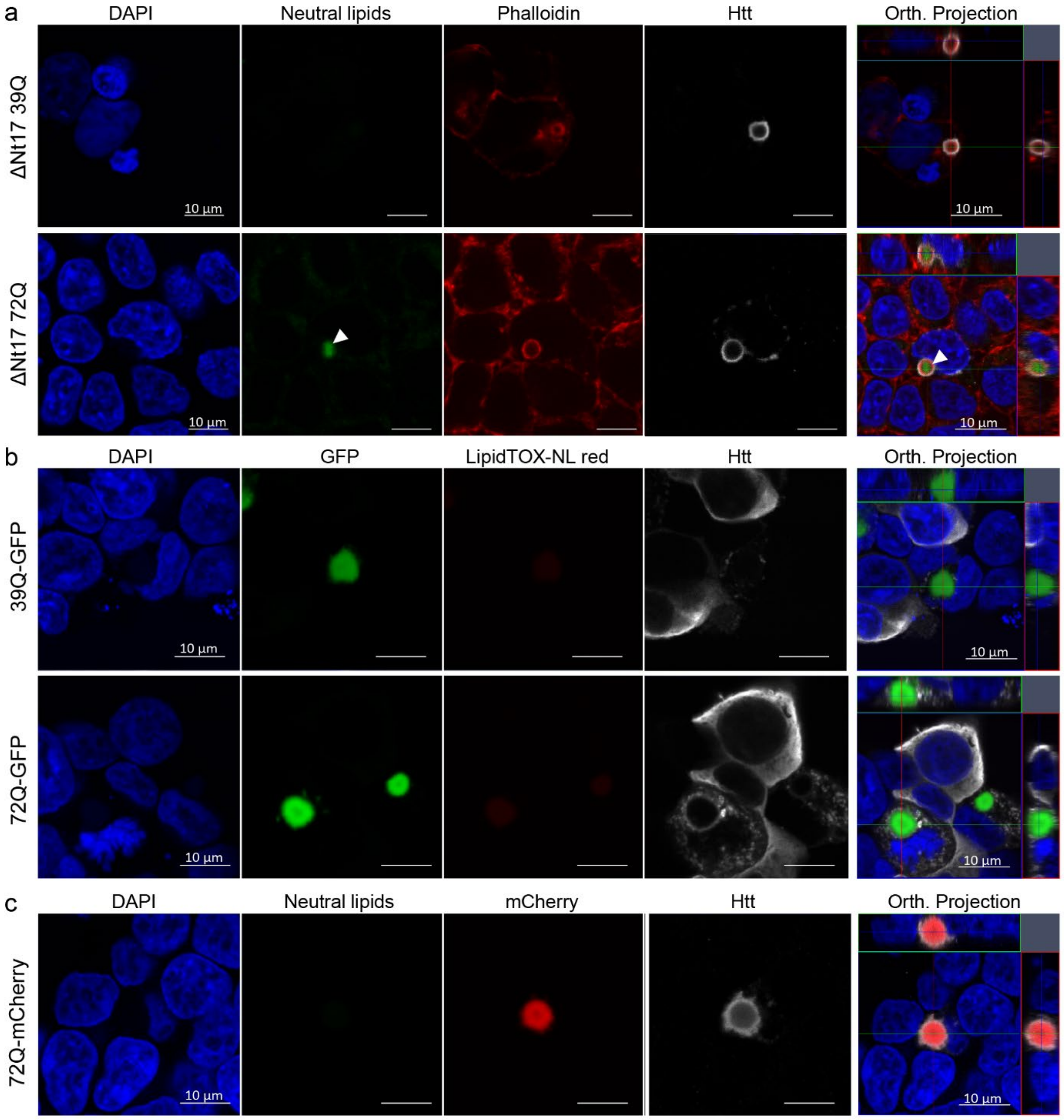

Supplementary Figure 10. **No neutral lipid enrichment was observed in Httex1-GFP cellular inclusions but only for Httex1 ΔNt17 72Q. a** Representative confocal image of Httex1 ΔNt17 39Q and 72Q inclusions 48 h post-transfection stained with the non-Polar BODIPY probe (493/503) targeting neutral lipids (green) shows a neutral lipid enrichment to the core of the inclusion for 72Q but not 39Q. Phalloidin (red) was used to stain the F-actin. White arrowheads indicate neutral lipid enrichment. Scale bars = 10 µm. **b** Representative confocal images of Httex1 39Q-GFP and Httex1 72Q-GFP inclusions 48 h post-transfection stained with the LipidTOX™ Red stain targeting neutral lipids (LipidTOX-NL red). **c** Representative confocal images of Httex1 72Q-mCherry inclusions 48 h post-transfection stained with the non-Polar BODIPY probe (493/503) targeting neutral lipids. No lipid enrichment was observed for Httex1-GFP or Httex1-mCherry cellular inclusions. The nucleus was stained with DAPI (blue) and Httex1 with MAB5492 primary antibody revealed by a secondary antibody coupled to Alexa 647 (grey). Orthogonal projections (Orth. Projection) were generated from a Z-stack through the selected cells. Scale bars = 10 µm.

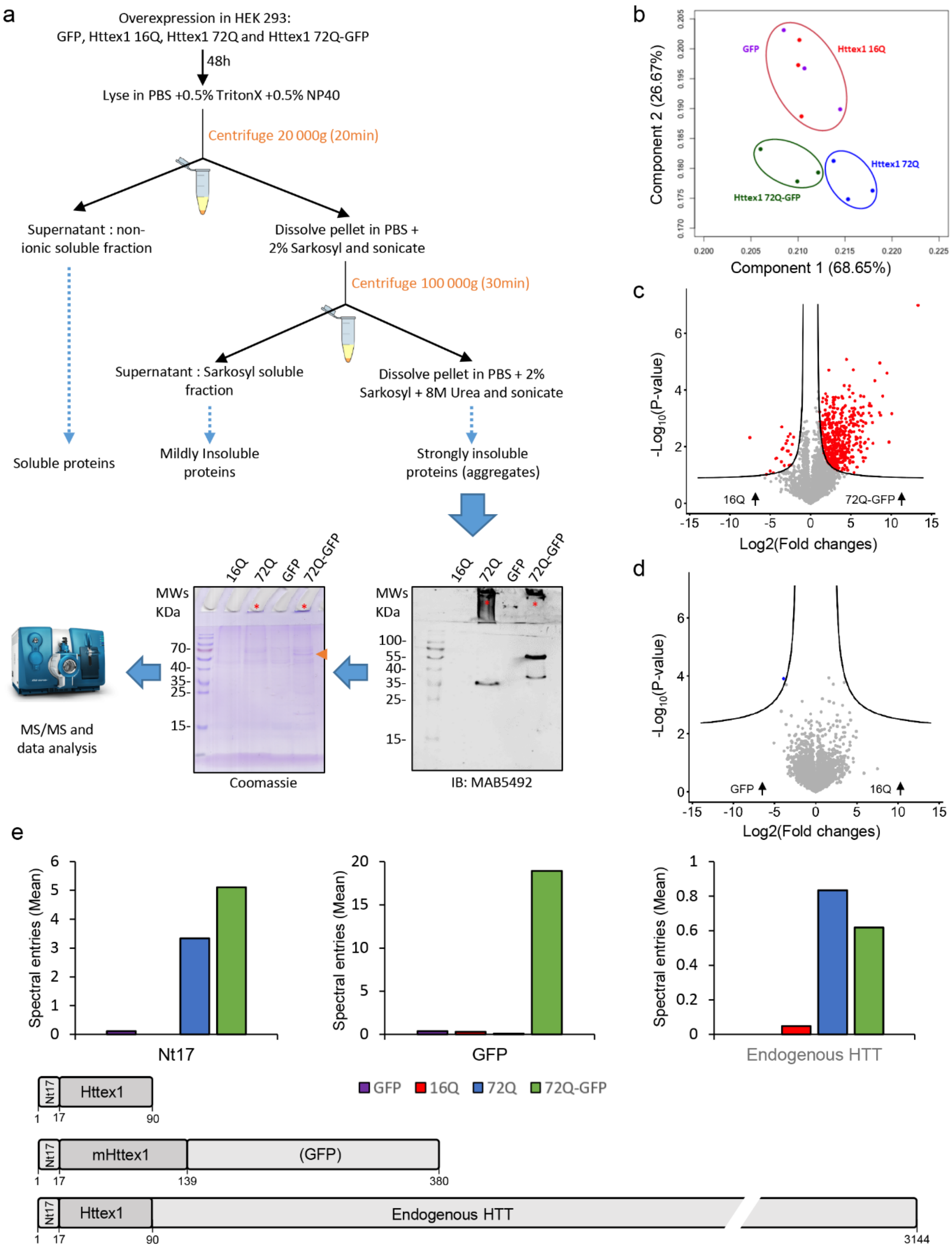

Supplementary Figure 11. **Detergent fractionation and proteomic analysis of Httex1 transfected in HEK cells. a** Overall workflow: HEK cells were transfected for 48 h with Httex1 and GFP indicated plasmids before detergent fractionation from mild to harsh solubilization to separate soluble Htt to aggregate species. Proteins from the last Urea soluble fraction containing Htt inclusions were separated on SDS-PAGE gel, followed by LC-MS/MS for protein identification and quantification of 3 independent experiments. Red stars indicate the presence of aggregates in the stacking gel. The orange arrow indicates the expected size of Httex1 72Q-GFP. Full scans and repeats are presented Supplementary Figure 31. **b** Principal component

analysis of the Urea soluble fraction shows 3 clusters: 1) Httex1 16Q and GFP (non-aggregated controls, red and purple), 2) Httex1 72Q-GFP (green), and 3) Httex1 72Q (blue). c-d Volcano plot with a false discovery rate (FDR) of 0.05 and S0 of 0.5 used to compare protein levels identified in the Urea soluble fraction. (**c**) The comparison of Httex1 72Q-GFP vs. Httex1 16Q showed a strong protein enrichment for Httex1 72Q-GFP. (**d**) Almost no significant differences were found in the comparison of the two negative controls Httex1 16Q and GFP. **e** Peptide detection (mean spectral entries of the 3 independent experiments) along the Httex1 (+/-GFP) and full-length HTT. The schematic representation of Htt fragments shows non-mutated Httex1 that corresponds to Httex1 16Q, mHttex1 corresponding to Httex1 72Q, or Httex1 72Q-GFP when fused to GFP at the C-terminus and the non-mutated full-length HTT that corresponds to the endogenous protein. The different sequences were divided into 4 segments: Nt17 domain, mHttex1 (not detected), GFP, and the full-length sequence of HTT over the first exon. The panel A was partially created with Biorender.

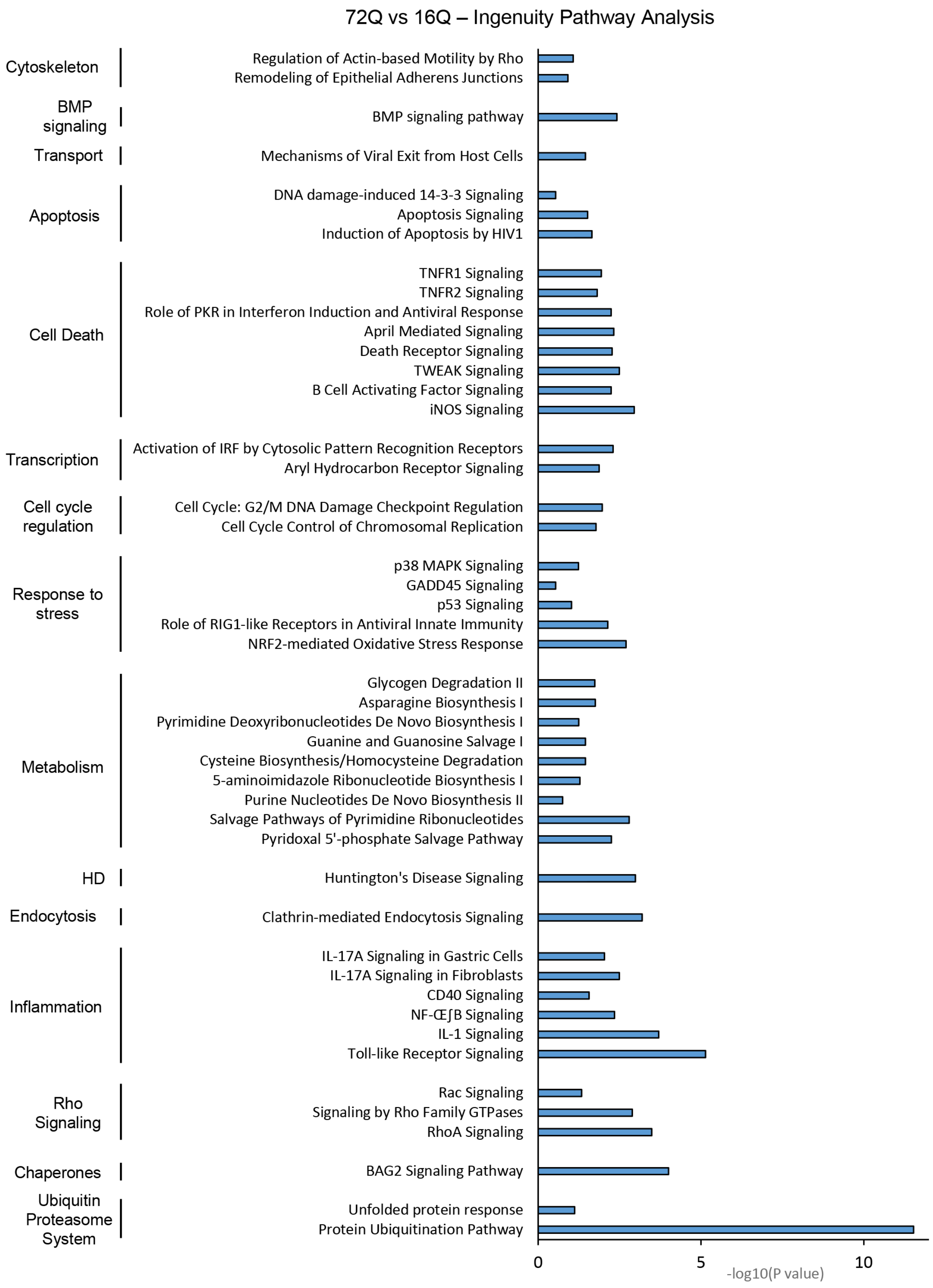

Supplementary Figure 12. **Ingenuity Pathway Analysis of Httex1 72Q vs. Httex1 16Q Urea soluble fraction reveals strong enrichment of the Ubiquitin-Proteasome System (UPS).** Canonical pathways enriched in the Urea soluble fraction of Httex1 72Q vs. Httex1 16Q extracted from the volcano plot (Figure 3a) of the quantitative proteomic using Ingenuity Pathway Analysis (IPA).

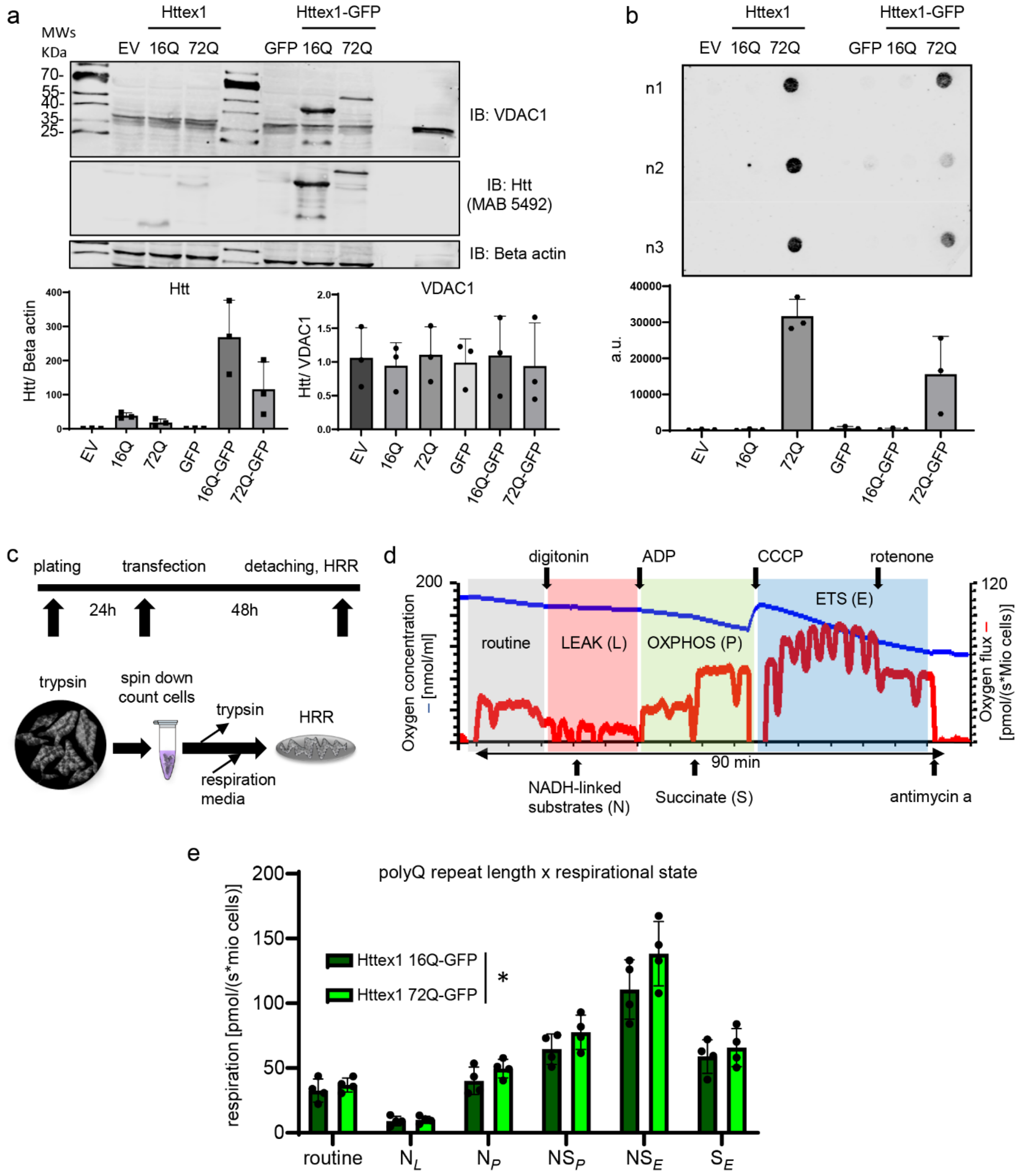

Supplementary Figure 13. **High-resolution respirometry (HRR) revealed respiration differences in cells transfected with Httex1 72Q-GFP compared to Httex1 16Q-GFP. a** Western Blot (WB) analyses of Httex1 transfected HEK cells in parallel with the HRR experiment. We quantified similar levels of the outer mitochondrial membrane protein VDAC1, indicating no decrease in mitochondrial density. Httex1 levels indicated higher Httex1 16Q(+/-GFP) levels compared to Httex1 72Q(+/-GFP) in the soluble fraction. Due to better transfection efficiency, Httex1-GFP constructs are expressed higher than the tag-free Httex1 constructs. Empty vector (EV). Data are presented as mean values +/- SD. Full scans and repeats are presented Supplementary Figure 32a-c. **b** Filter trap analyses of Httex1 transfected HEK cells in parallel with the HRR experiment. Only Httex1 72Q(+/-GFP) were detected on the filter trap after loading of the SDS-insoluble fraction, indicating the formation of large SDS-insoluble aggregates. Empty vector (EV). Data are presented as mean values +/- SD. Full scan with

repeats are presented Supplementary Figure 32d. **c** Experimental setup of HRR experiments. Cells were transfected with indicated constructs 24 h after plating in 4 independent experiments. 48 h after transfection, cells were gently detached and HRR was performed in respiration media (MIR05). **d** After the measurement of routine respiration, cells were chemically permeabilized by digitonin. Different respirational states were subsequently induced using a substrate-uncoupler-inhibitor titration (SUIT) protocol. **e** Routine respiration, NADH-driven, or complex 1-linked respiration after the addition of ADP (OXPHOS state) (NP), NADH- and succinate driven, or complex 1 and 2-linked respiration in the OXPHOS state (NSP), and in the uncoupled electron transport system (ETS) capacity (NSE), as well as succinate driven, or complex 2-linked respiration in the ETS state (SE) were assessed. Httex1 72Q-GFP significantly increased the respiration compared to Httex1 16Q-GFP. n=4 biologically independent experiments. Data are presented as mean values +/- SD. Two-way ANOVA showing a significant interaction between the polyQ repeat length and the respirational states (p-value=0.0305). *P < 0.05, **P < 0.005, ***P < 0.001. The panel C was partially created with Biorender.

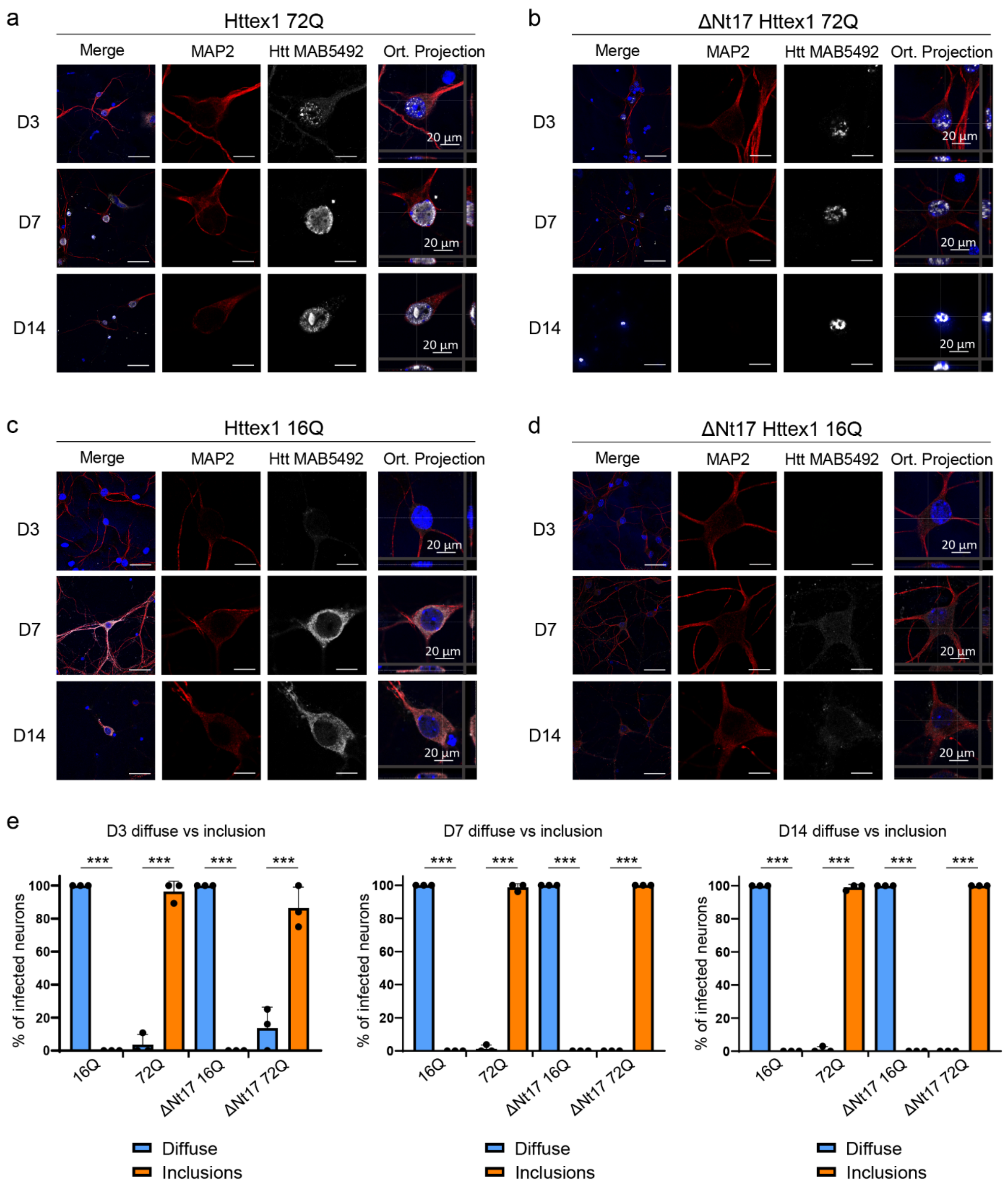

Supplementary Figure 14. **Most of the neurons overexpressing Httex1 72Q show the presence of nuclear aggregates.** Representative images of Httex1 expression of **a** Httex1 72Q; **b** ΔNt17 Httex1 72Q; **c** Httex1 72Q; **d** ΔNt17 Httex1 72Q; detected by ICC staining combined with confocal imaging in primary cortical neurons at 3 (D3), 7 (D7) and 14 (D14) days after lentiviral transduction. Httex1 was detected with the MAB5492 antibody (grey) and the neurons with the MAP2 antibody (red). The nucleus was counterstained with DAPI (blue). Scale bar = 20 µm. **e** Image-based quantification of neurons expressing Httex1 as diffuse protein or containing Httex1 inclusions at D3, D7 and D14. The graphs represent the mean ± SD of 3 independent experiments. Statistical analysis: One-way ANOVA followed by a Tukey honest significant difference [HSD] post hoc test was performed. P-value<0.0001 (left panel), p-value<0.0001 (middle panel) and p-value<0.0001 (right panel) and *P < 0.05, **P < 0.005, ***P < 0.001 for multiple comparisons.

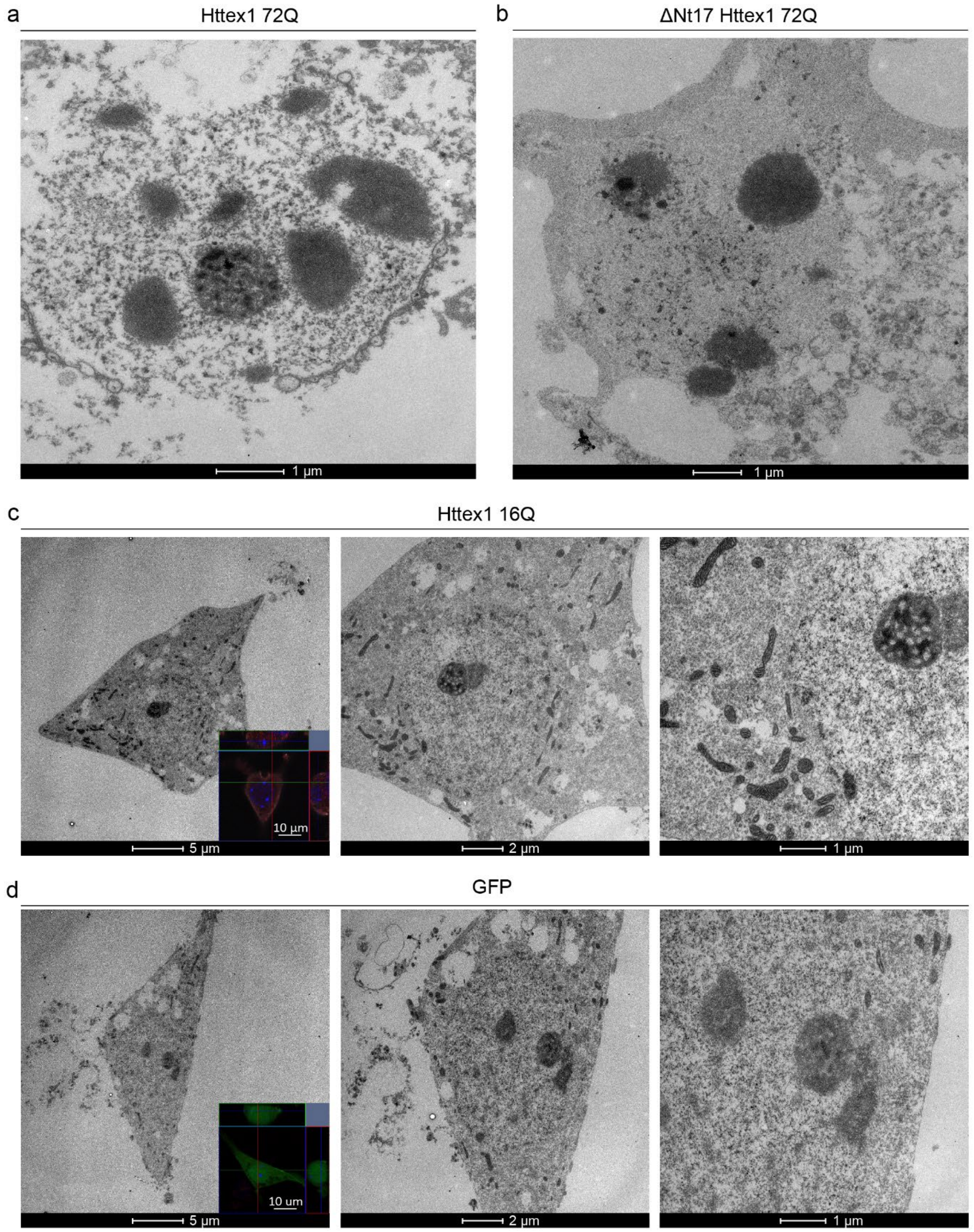

Supplementary Figure 15. **Representative electron micrographs of primary neurons expressing Httex1 showing inclusion formation by Httex1 72Q and ΔNt17 Httex1 72Q, but not 16Q, or GFP.**

Representative electron micrographs of **a** Httex1 16Q and **b** GFP, transduced neuron selected by fluorescence using CLEM. The fluorescence images (insets) were acquired by confocal imaging. Httex1 was detected with the MAB5492 antibody, the neurons with the MAP2 antibody (red) and the GFP in green. The nucleus was counterstained with DAPI (blue). **c** Representative electron micrograph of Httex1 72Q transduced neurons at low magnification showing disruption of the nuclear envelop and cellular integrity from close up image Figure 6a

(right panel). **d** Representative electron micrograph of ΔNt17 Httex1 72Q transduced neurons. Scale bars = 500 nm for the EM images and 50 μm for the fluorescent images (insets).

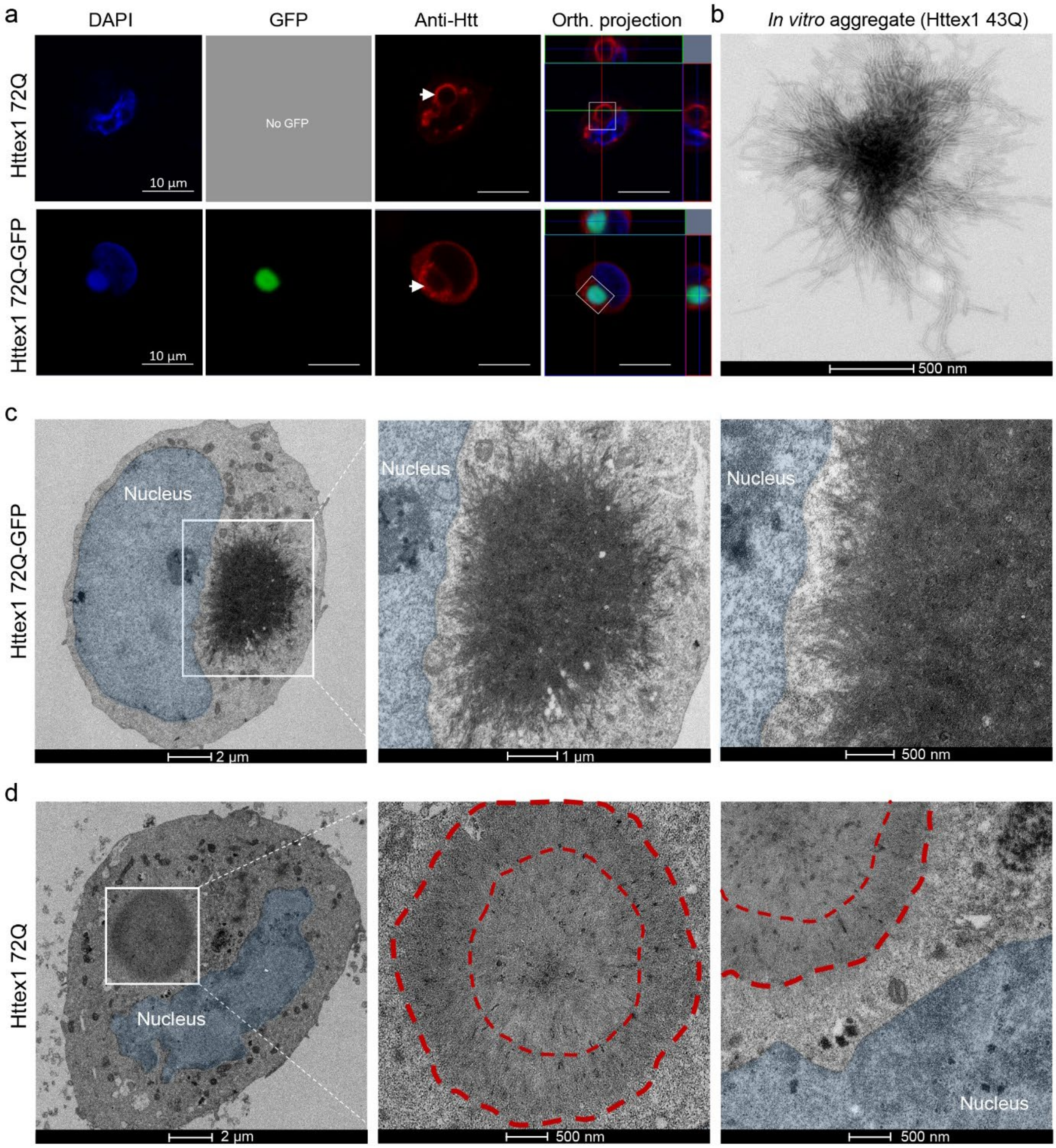

Supplementary Figure 16. **Correlative light- and electron microscopy (CLEM) of cells transfected with Httex1 72Q and Httex1 72Q-GFP. a** Confocal images of Httex1 72Q and Httex1 72Q-GFP, 48 h after transfection in HEK cells. Httex1 expression (red) was detected using a specific primary antibody against the N-terminal part of Htt (MAB5492) or GFP (green), and the nucleus was stained with DAPI (blue). Scale bars = 10 μm. The same cell was then processed for EM. **b** Representative image of in vitro aggregate of Httex1 43Q assessed by EM. **c** Electron micrograph of the transfected cell by Httex1 72Q-GFP previously imaged by confocal (A, bottom panels). Magnified micrographs of the inclusion (white square) and magnification close to the nuclear membrane are displayed on the middle and right-hand panels. **d** Electron micrographs of the transfected cell by Httex1 72Q previously imaged by confocal (A, upper panels). Magnified micrographs of the inclusion (white square) and magnification close to the nuclear membrane are displayed on the middle and right-hand panels. Scale bars = 2 μm, 1 μm, or 500nm as indicated below the micrographs.

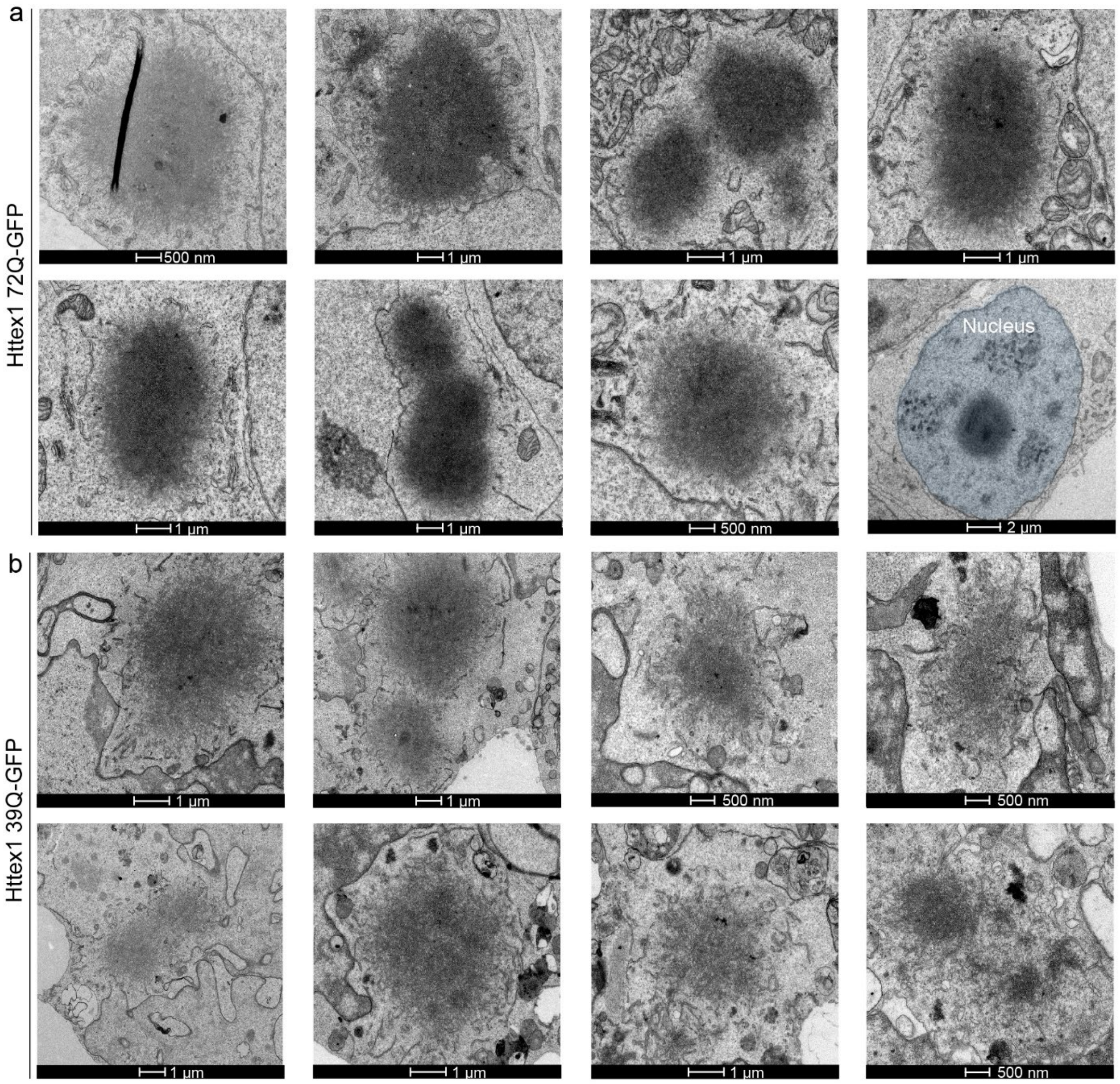

Supplementary Figure 17. **Ultrastructural characterization of Httex1 72Q-GFP and Httex1 39Q-GFP inclusions. a** 8 representative electron micrographs of Httex1 72Q-GFP inclusions formed in HEK cells 48 h post-transfection. **b** 8 representative electron micrographs of Httex1 39-GFP inclusions formed in HEK cells 48 h post-transfection. The nucleus was highlighted in blue. Scale bars = 1 µm or 500 nm as indicated below the micrographs.

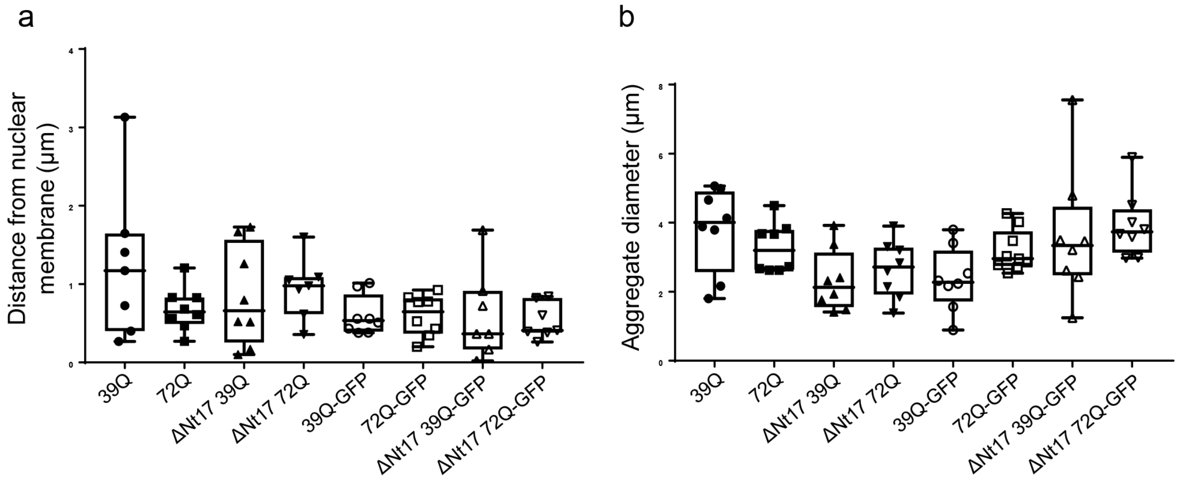

Supplementary Figure 18. **Subcellular localization and size of Httex1 inclusions based on electron micrographs.** The distance of the inclusion from the nuclear membrane and the inclusion diameter was quantified from electron micrographs from Figures S3, S8, S17, and S20. **a** Distance of the inclusion from the nuclear membrane. **b** Httex1 inclusion diameter. The boxes of the boxplots extend from the 25th to 75th percentiles, the whiskers represent the minimum and maximum values and the middle line represents the median. A minimum of n=7 inclusions were examined over 3 independent experiments. No statistical differences were measured between the different conditions.

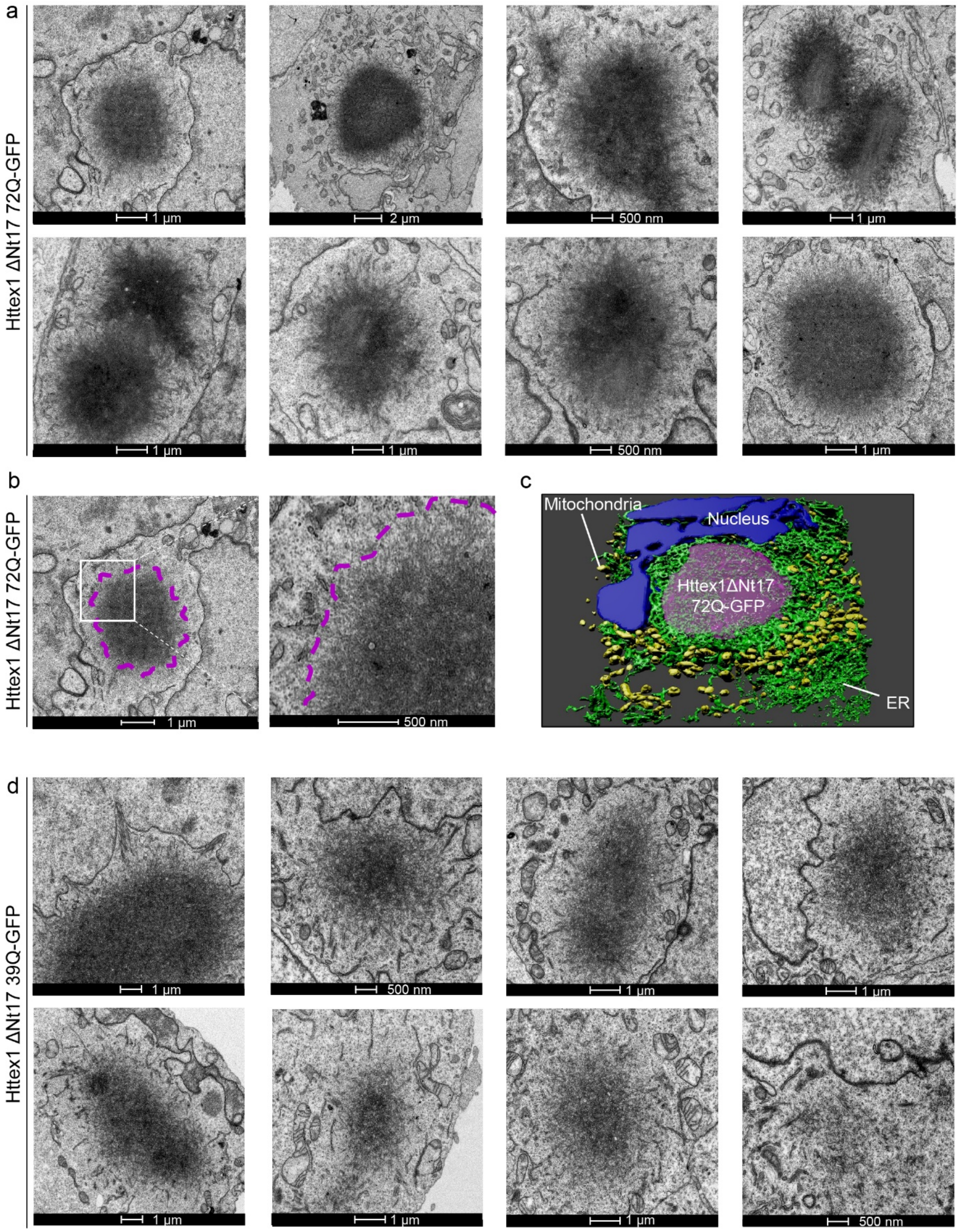

Supplementary Figure 19. **EM and 3D models of cellular Httex1 39Q-GFP and 72Q-GFP inclusions show ER and mitochondria in their periphery. a-b** Representative electron micrographs of Httex1 39Q-GFP (a) and Httex1 72Q-GFP (b) inclusions formed after 48 h expression in HEK cells. Higher magnifications (white square) are represented in the right-hand panels. Dashed lines delimit the inclusions. Orange arrowheads: internalized membranous structures. Green arrowheads: mitochondria. More electron micrographs of Httex1 72Q-GFP (Supplementary Figure 15a) and Httex1 39Q-GFP (Supplementary Figure 15b) were acquired. Scale bars = 1 µm (left-hand panel) and 500 nm (right-hand panels). **c-d** 3D models of Httex1 39Q-GFP (**c**) and Httex1 72Q-GFP (**d**) inclusions (top views). Httex1-

GFP inclusions (purple), ER membranes (green), nucleus (blue), and mitochondria (yellow). Scale bars = 2 μm.

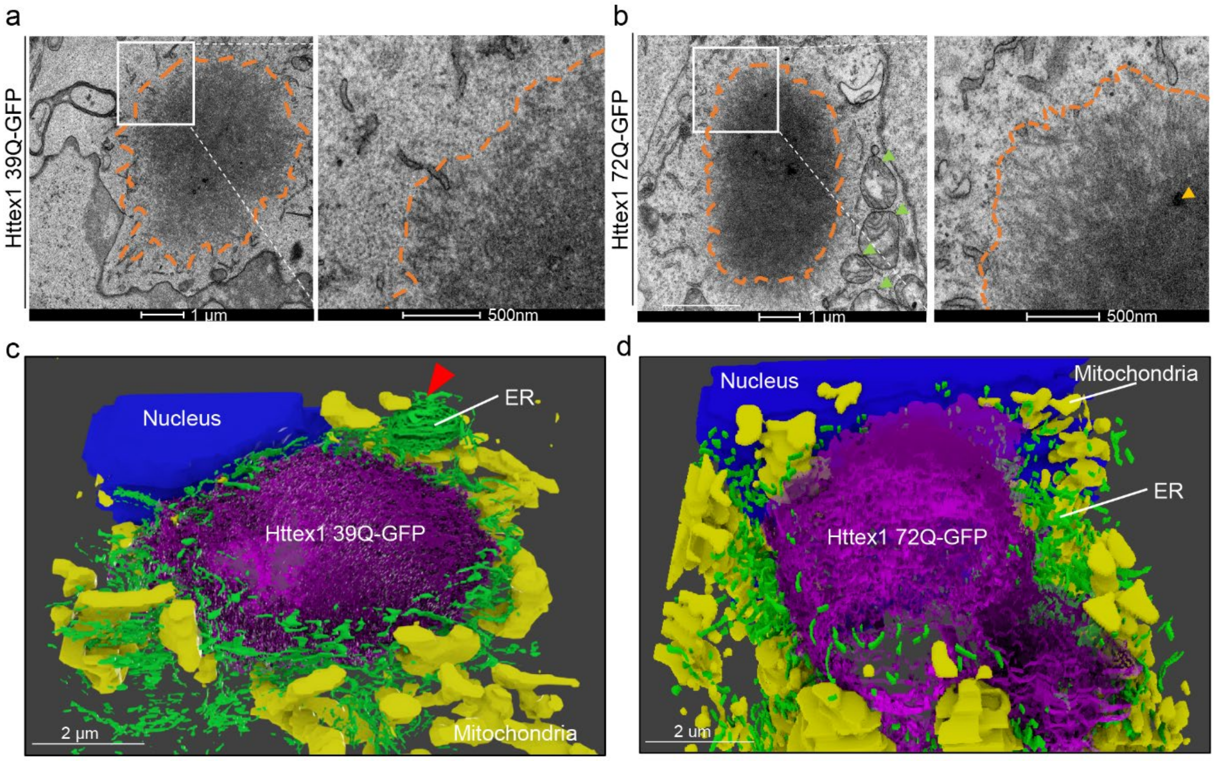

Supplementary Figure 20. **Ultrastructural characterization of Httex1 ΔNt17 72Q-GFP and Httex1 ΔNt17 39Q-GFP inclusions. a** 8 representative electron micrographs of Httex1 ΔNt17 72Q-GFP inclusions formed in HEK cells 48 h post-transfection. **b** Httex1 ΔNt17 72Q-GFP cellular inclusion and higher magnification (white square) are displayed in the right-hand panel. Dashed lines delimit the inclusion. **c** 3D model of Httex1 ΔNt17 72Q-GFP cellular inclusion (top view). Httex1 ΔNt17 72Q-GFP inclusion (purple), ER membranes (green), nucleus (blue), and mitochondria (yellow). **d** 8 representative electron micrographs of Httex1 ΔNt17 39-GFP inclusions formed in HEK cells 48 h post-transfection. Scale bars = 2 μm, 1 μm, or 500 nm as indicated below the micrographs.

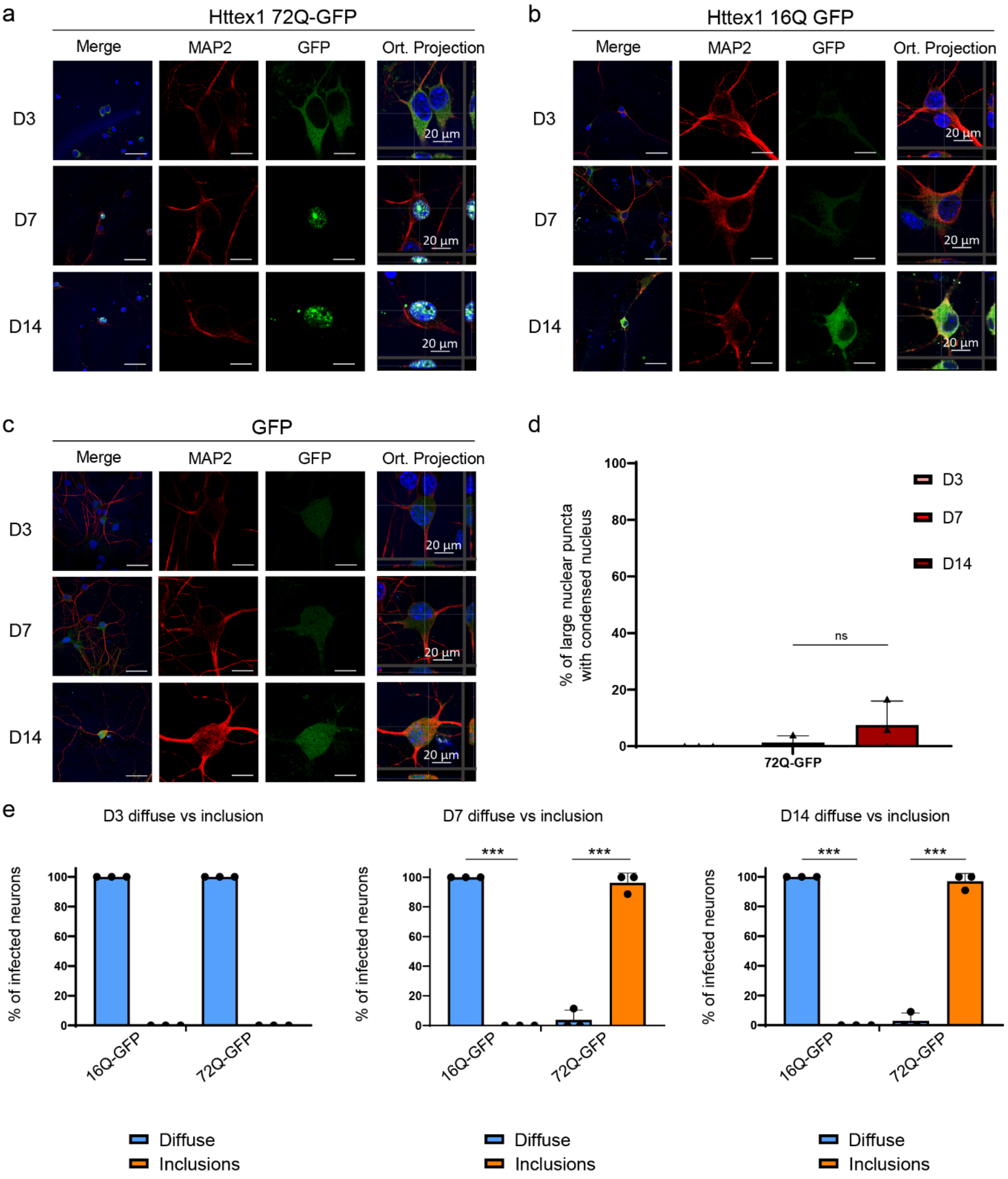

Supplementary Figure 21. **Neurons overexpressing Httex1 72Q-GFP show the presence of nuclear aggregates only from D7.** Representative images of Httex1 expression of Httex1-GFP and GFP expression of **a** Httex1 72Q-GFP; **b** ΔNt17 Httex1 72Q-GFP; **c** GFP; detected by ICC staining combined with confocal imaging in primary cortical neurons at 3 (D3), 7 (D7) and 14 (D14) days after lentiviral transduction. Httex1 was visualized with the GFP, and the neurons were detected with the MAP2 antibody (red). The nucleus was counterstained with DAPI (blue). Scale bar = 20 µm. **d** Image-based quantification of neurons containing a large nuclear inclusion with a nuclear condensation. **e** Image-based quantification of neurons expressing Httex1 as diffuse protein or containing Httex1 inclusions at D3, D7 and D14. **d-e**. The graphs represent the mean ± SD of 3 independent experiments. Statistical analysis: One-way ANOVA followed by a Tukey honest significant difference [HSD] post hoc test was performed. P-value<0.0001 (middle panel) and p-value<0.0001 (right panel) and *P < 0.05, **P < 0.005, ***P < 0.001 for multiple comparisons.

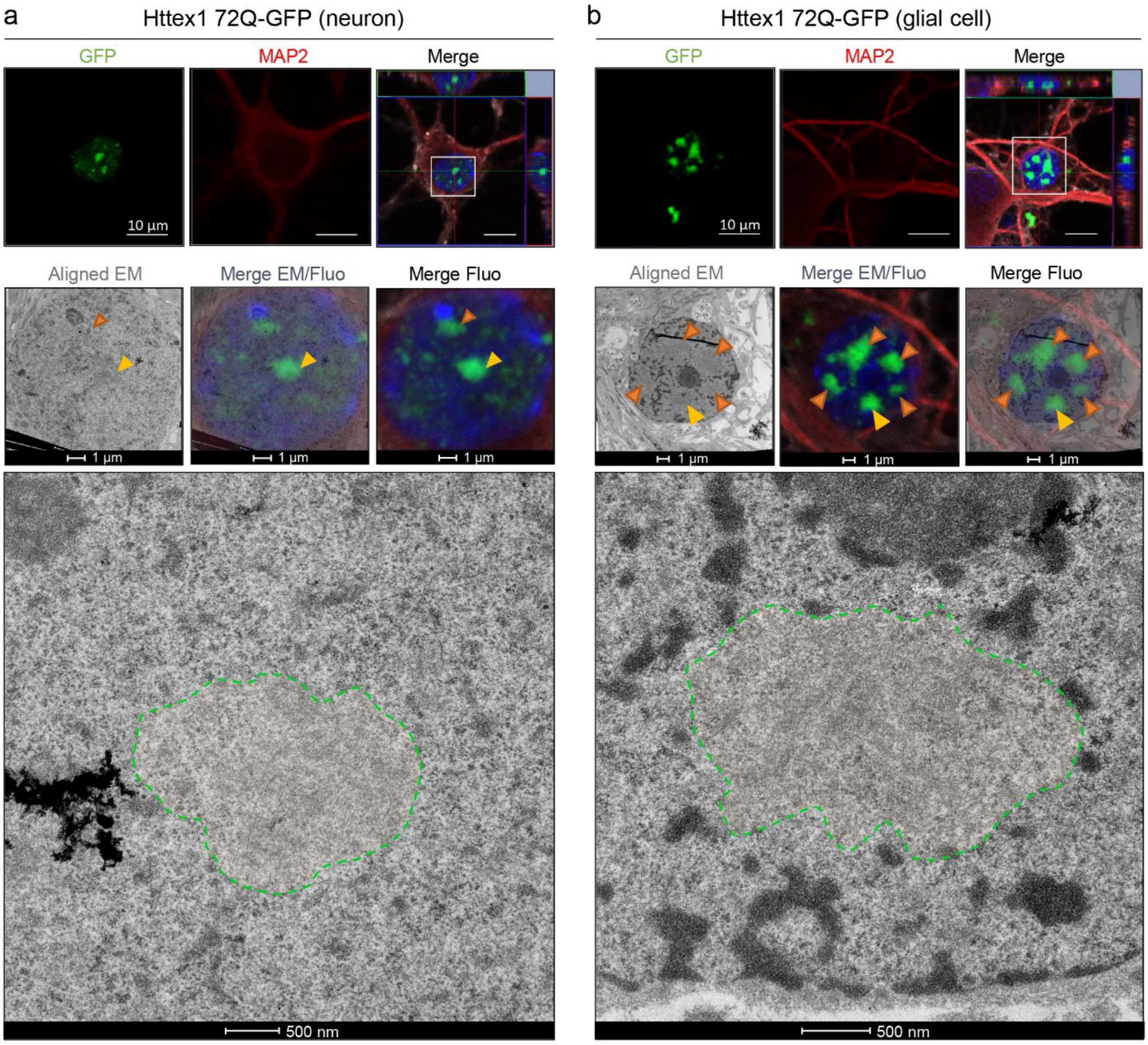

Supplementary Figure 22. **CLEM analysis of the ultrastructure properties of Httex1 72Q-GFP formed in glial cells and neurons**. Seven days post-transduction, a selected neuron (**a**) and glial cell (**b**) were fixed and subjected to ICC staining in order to image and localize Httex1 72Q-GFP inclusions by confocal microscopy (upper panels). Httex1 was detected with GFP, the nucleus was counterstained with DAPI (blue), and the neurons were detected using MAP2 antibodies (red). Scale bars = 10 µm. Fluorescence images allowed the selection of the cell of interest and to correct the alignment of the inclusions with the electron micrographs (middle panels, orange arrowheads: Httex1 72Q-GFP nuclear inclusions; yellow arrowheads: selected inclusion). Selected inclusions could be successfully segmented (green-dotted lines, lower panels. Scale bars = 500 nm.

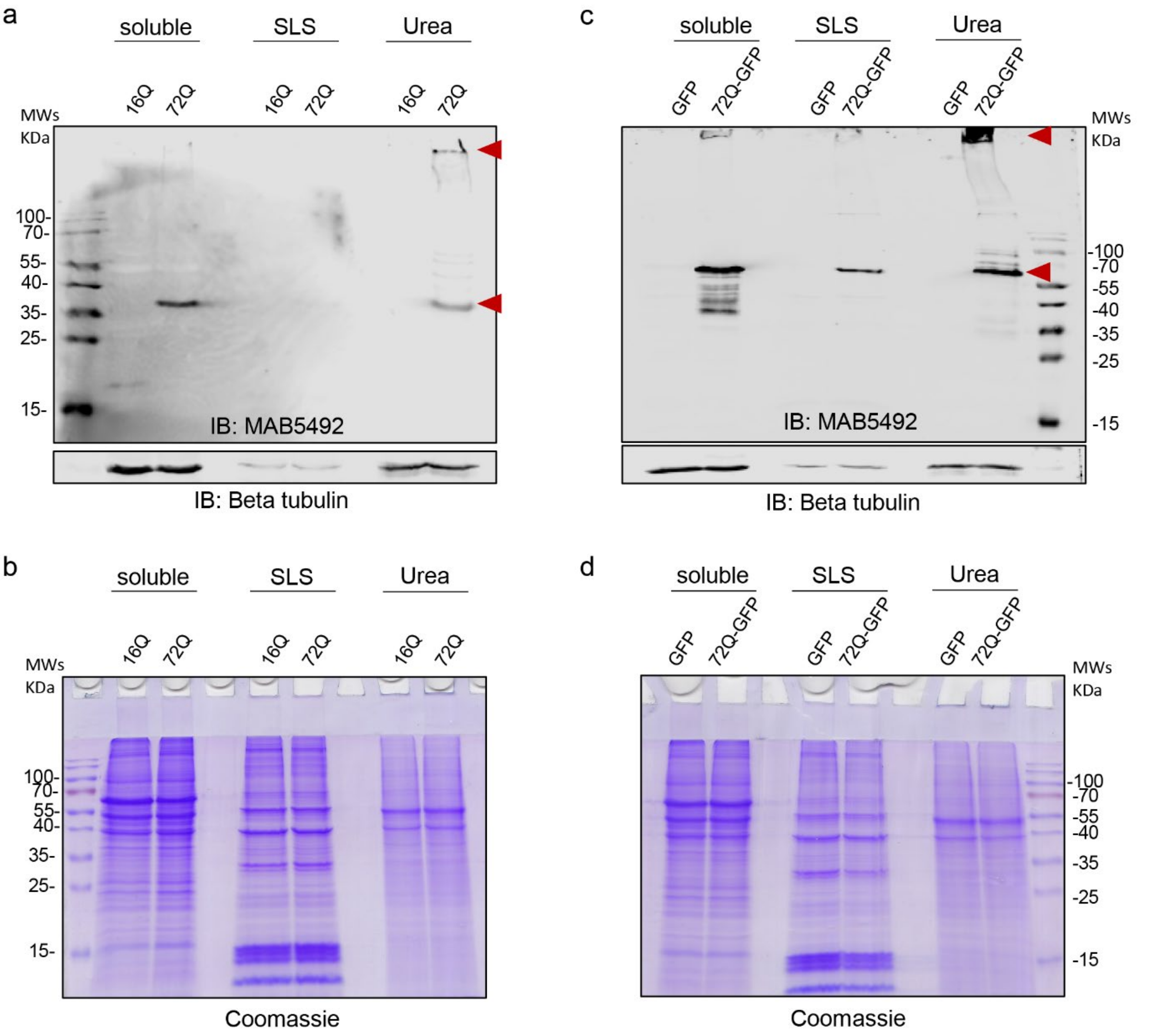

Supplementary Figure 23. **The enrichment of Httex1 aggregates in the soluble urea fraction of primary neurons was confirmed by Western Blot analysis.** Cell fractionation from transduced primary neurons was performed as detailed in Supplementary Figure 11a. Httex1 16Q and Httex1 72Q (**a-b**) or GFP and Httex1 72Q-GFP (**c-d**) protein expression levels in the different detergent fractions were assessed by WB (a, c) and Coomassie staining (b, d). The aggregation-prone Httex1 constructs (Httex1 72Q and Httex1 72Q-GFP) could successfully be detected in the last aggregate Urea fraction (red arrowheads) but not the controls Httex1 16Q and GFP. Httex1 was detected by WB using antibody MAB5492 and Beta tubulin was used as the loading control. Source data are provided as a Source Data file.

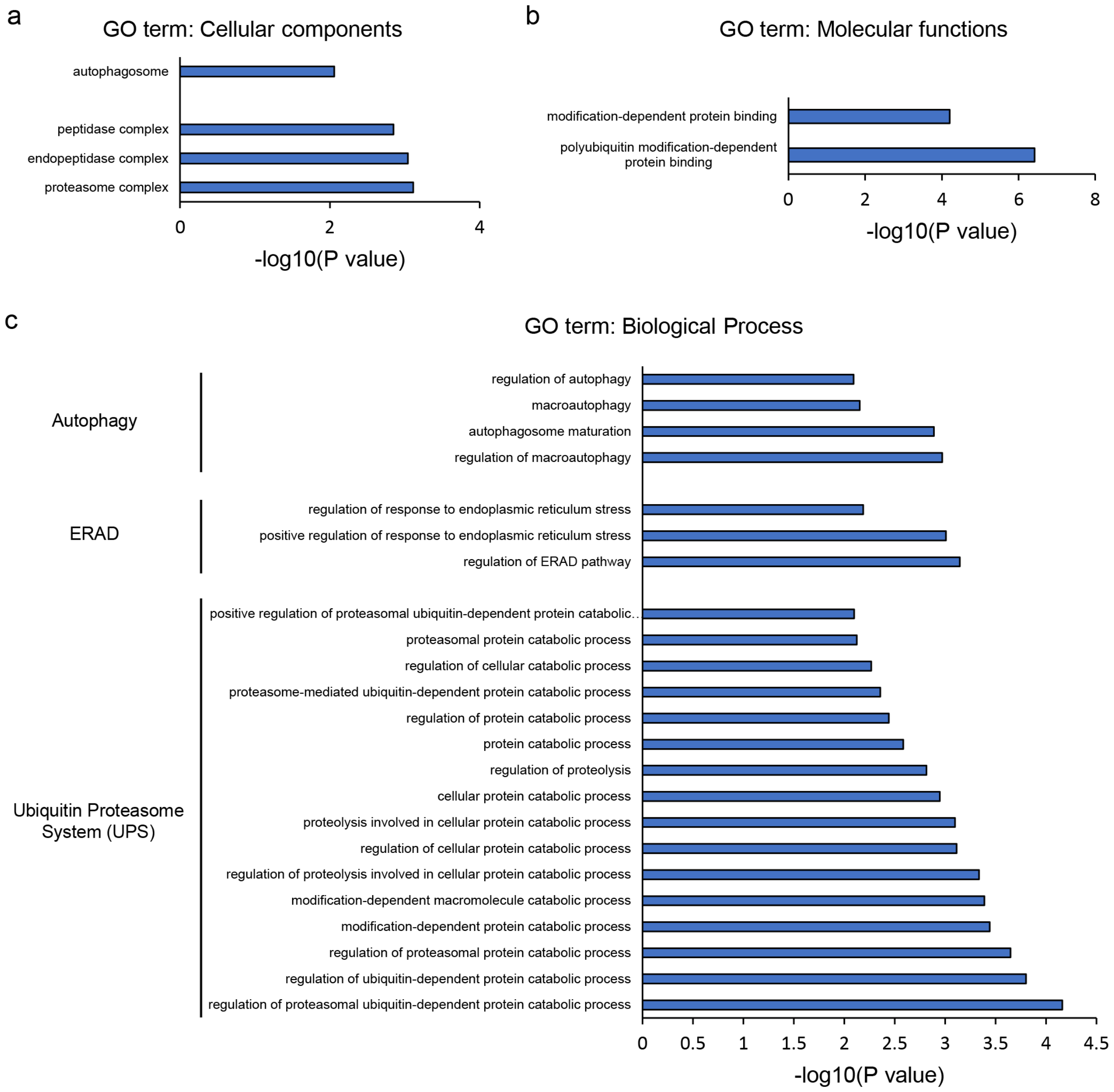

Supplementary Figure 24. **Proteomic analysis of Httex1 72Q vs. Httex1 16Q Urea soluble fraction revealed strong enrichment of the Ubiquitin-Proteasome System.** Cellular components (**a**), Molecular functions (**b**) and biological processes (**c**) enriched in the Urea soluble fraction of Httex1 72Q vs. Httex1 16Q extracted from the volcano plot (p-value<0.01) (Figure 8f). Analyses were performed using Gene Ontology (GO) enrichment analyses determined by DAVID analysis (–$\log_{10}$(p-value) >1).

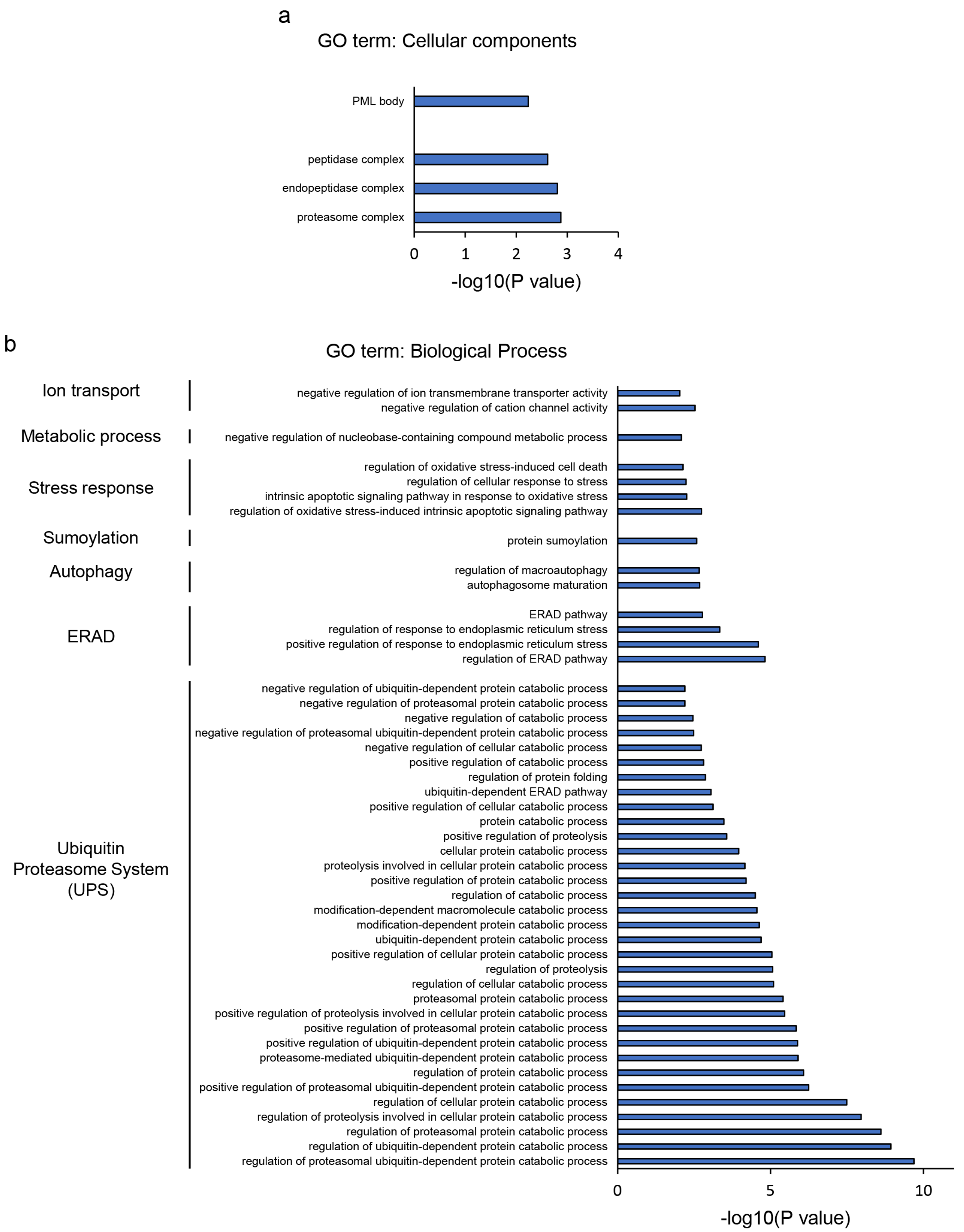

Supplementary Figure 25. **Proteomic analysis of Httex1 72Q-GFP vs. GFP Urea soluble fraction revealed strong enrichment of the Ubiquitin-Proteasome System.** Cellular components (**a**) and biological processes (**b**) enriched in the Urea soluble fraction of Httex1 72Q vs. Httex1 16Q extracted from the volcano plot (p-value<0.01) (Figure 8h). Analyses were performed using Gene Ontology (GO) enrichment analyses determined by DAVID analysis (–$\log_{10}$(p-value) >1).

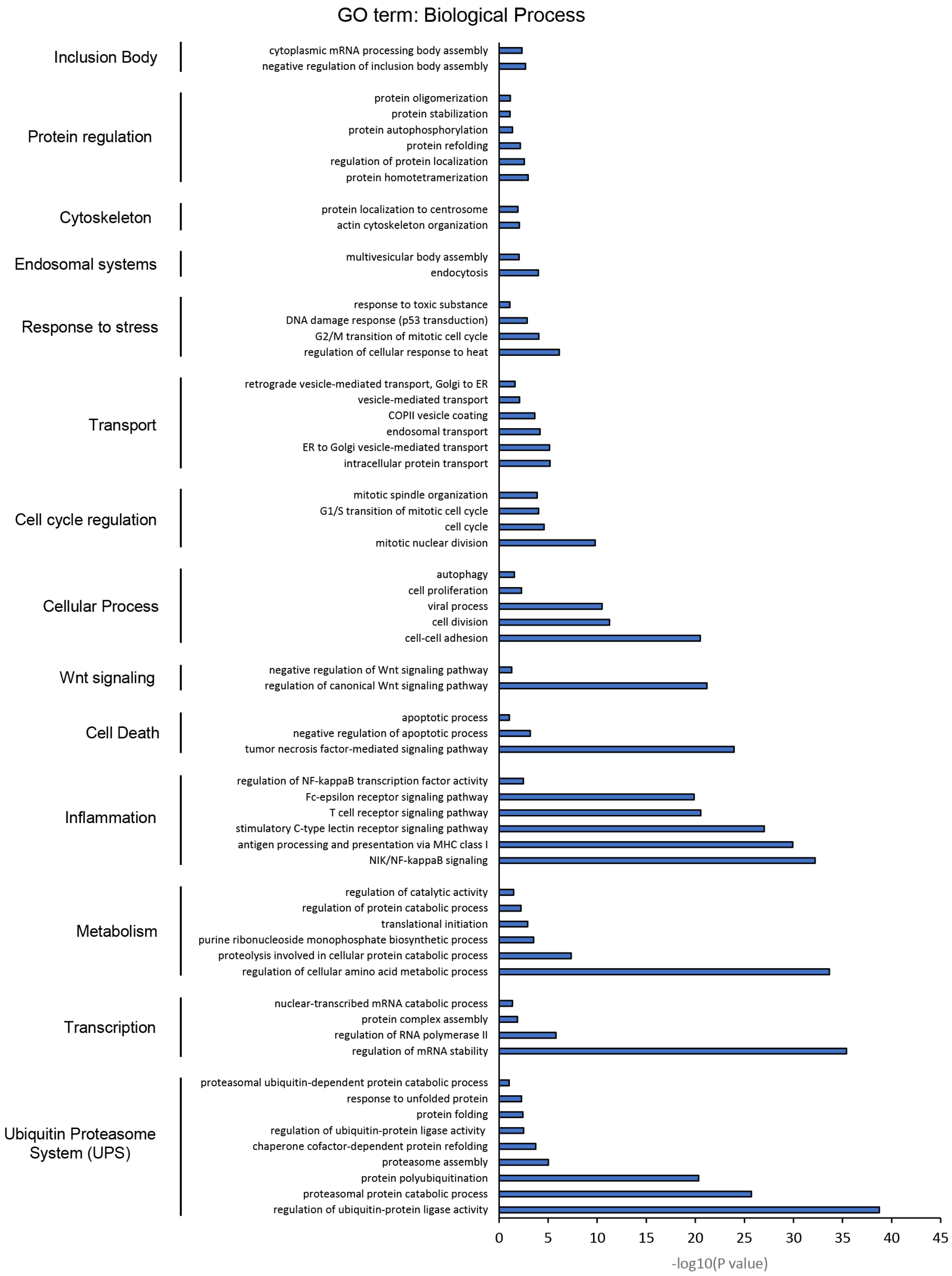

Supplementary Figure 26. **Proteomic analysis of Httex1 72Q-GFP vs. GFP Urea soluble fraction from HEK cells.** Classification of the proteins significantly enriched in the Urea soluble fraction of HEK cells overexpressing Httex1 72Q or Httex1 16Q and extracted from the volcano plot (p-value<0.01) (Figure 3a). Analyses were performed using Gene Ontology (GO) enrichment analyses determined by DAVID analysis (–$\log_{10}$(p-value) >1).

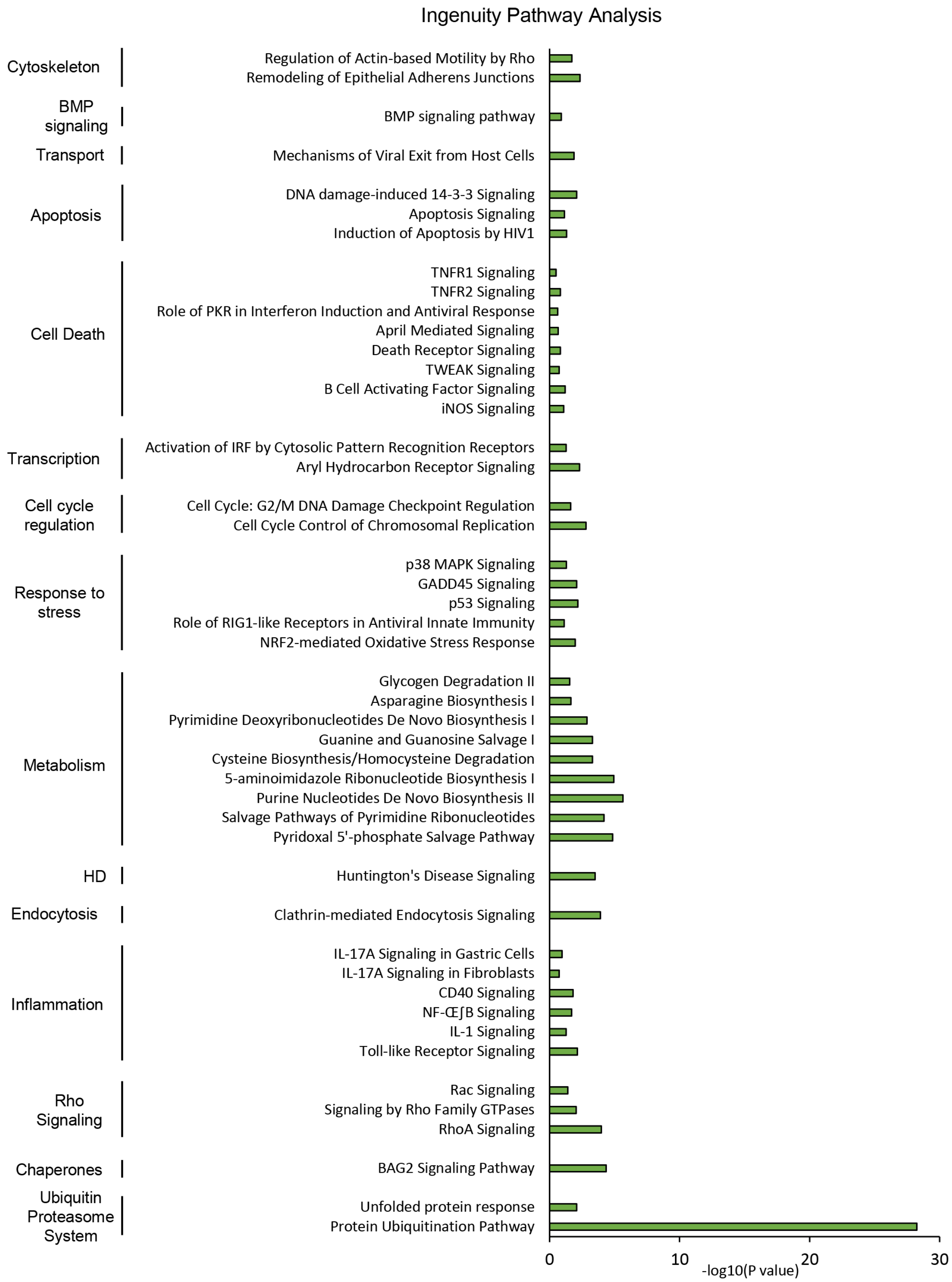

Supplementary Figure 27. **Ingenuity Pathway Analysis of Httex1 72Q-GFP vs. GFP Urea soluble fraction revealed strong enrichment of the Ubiquitin-Proteasome System (UPS).** Canonical pathways enriched in the Urea soluble fraction of Httex1 72Q-GFP vs. Httex1 GFP extracted from the volcano plot in Figure 9a. Analyses were performed using Ingenuity Pathway Analysis (IPA).

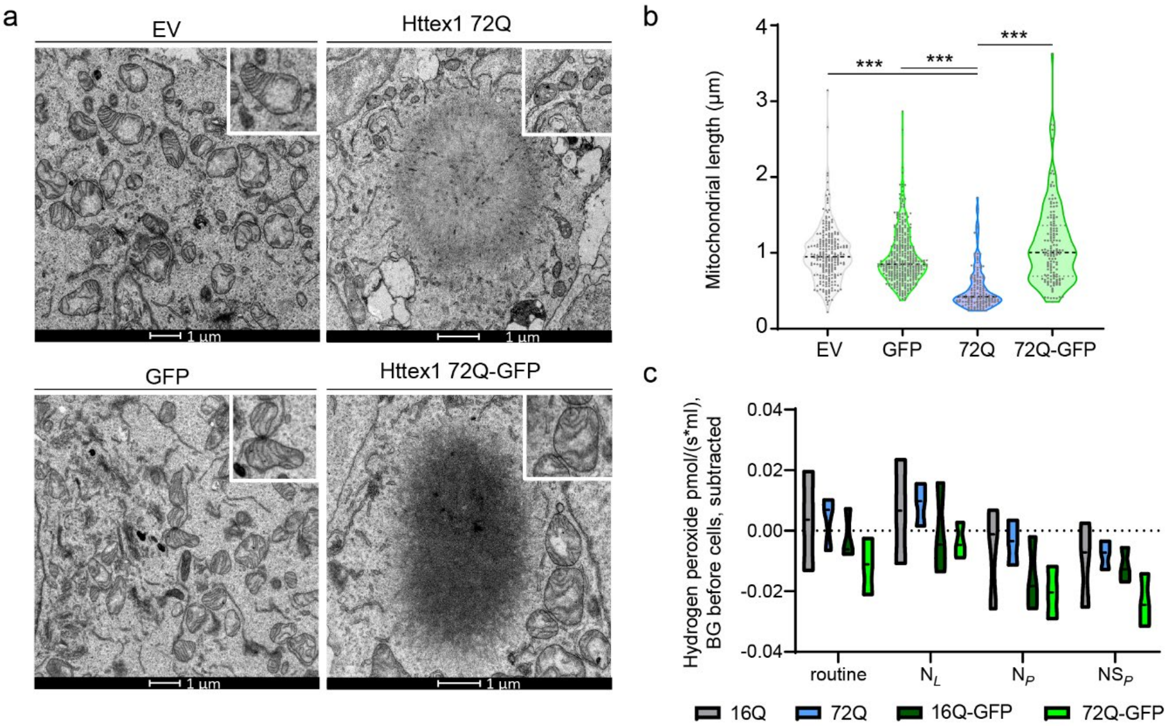

Supplementary Figure 28. **The formation of 72Q Httex1 inclusions induces mitochondrial alterations. a** Electron micrographs of mitochondria in HEK cells overexpressing empty vector (EV), Httex1 72Q, GFP or Httex1 72Q-GFP. The insets depict higher magnification of the mitochondria found at the periphery of Httex1 inclusions or representative in EV and GFP controls. Scale bars = 1 µm. **b** Measurement of the mitochondrial length reveals a significant reduction of the size of the mitochondria profile located in the proximity of the inclusions. Statistical analysis: Kruskal-Wallis test was performed resulting to a p-value<0.0001 and *P < 0.05, **P < 0.005, ***P < 0.001 for multiple comparisons. **c** Mitochondrial reactive oxygen species (ROS) were measured in HEK cells overexpressing Httex1 16Q, Httex1 16Q-GFP, Httex1 72Q or Httex1 72Q-GFP for 48 h. The produced mitochondrial ROS were measured using Amplex red fluorometry (superoxide was transformed by superoxide dismutase to detectable levels of hydrogen peroxide). Using a two-way ANOVA, no significant differences in mitochondrial ROS levels were detected in the HEK cells overexpressing Httex1 72Q, compared to Httex1 72Q-GFP (p-value=0.3792). The violin plot represents the median, minimum and maximum values of three independent experiments.

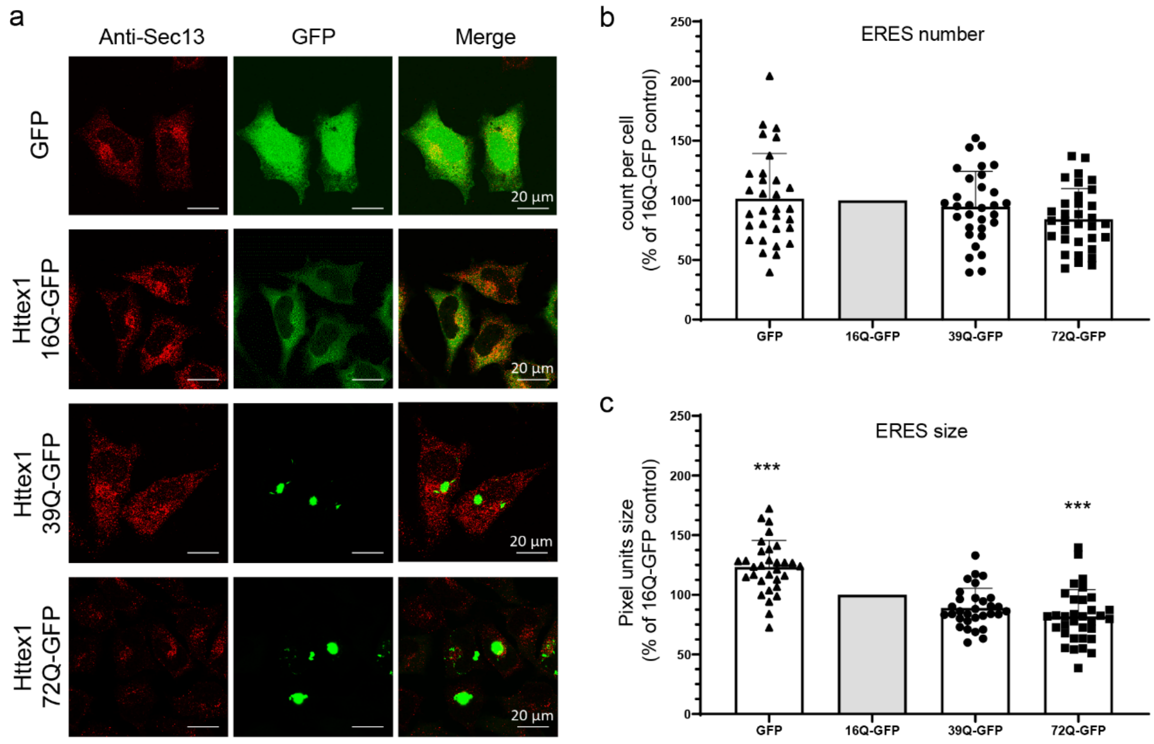

Supplementary Figure 29. **Httex1-GFP inclusion formation induces the size reduction of ER-exit sites. a** Representative confocal images of HeLa cells transfected with Httex1 16Q-GFP, 39Q-GFP or 72Q-GFP or GFP (as the negative control). Cells were fixed 48 h after transfection and immunostained. Httex1-GFP or GFP was detected by fluorescence, and ER exit sites were detected using the Sec13 antibody (red). Scale bars = 20 µm. **b-c** ERES number (**b**) and size (**c**) quantifications from confocal imaging were performed using FIJI. The graphs represent the mean ± SD of three independent experiments presented as the relative percentage of the Httex1 16Q-GFP control. One-way ANOVA followed by Tukey honest significant difference [HSD] post hoc test was performed. P-value=0.0434 (top panel) and p-value<0.0001 (bottom panel) and *P < 0.05, **P < 0.005, ***P < 0.001 for multiple comparisons.

Specific features	Httex1 72Q	Httex1 72Q-GFP
Actin F at the periphery of inclusions	+++	-
Ring detection of Htt Ab	+++	++ (+center faintly)
Core/shell organization	+++	-
Thickness and spacing of fibrils in periphery	Thin fibrils and more spacing than the core	Thick fibrils, more inter-space compare to tag-free
polyQ influence on ultrastructure	Yes (lose core/shell organization)	No
Recruitment of membranous organelles	++ in core and +++ in periphery	Few but interactions at the periphery
Nuclear inclusions	Lose core/shell arrangement and the recruitment of membranous organelles<br>No interactions with nuc. mb.	No overall change in morphology<br><br>No interactions with nuc. mb.
Lipids	++ Neutral lipids in core specific for 72Q not 39Q	-
Mitochondrial morphology	Loss of cristae	normal
Fragmentation of mitochondrial profile	++	-
Mitochondrial respiration	++	+
ER impact	++ ERES modulation	+ ERES modulation
Main pathways proteomic	Endolysosome, UPS, cytoskeleton, nucleoplasm, ER to Golgi transport	Endolysosome, UPS, cytoskeleton, nucleoplasm, ER to Golgi transport, mitochondria
Differences in proteomic pathways	Infection/inflammation, mRNA stability	UPS more enriched, mitochondria, metabolism

Supplementary Figure 30. **Tag-free and GFP Httex1 cellular inclusions reveal distinct features in HEK cells.** The table summarizes the key distinct features between Httex1 72Q and Httex1 72Q-GFP at the cellular, ultrastructural, proteomic composition and functional levels. Confocal analysis revealed a ring-like detection of Httex1 inclusions by Htt antibodies and colocalized with filamentous actin only for tag-free Httex1 72Q. Our results demonstrated that the core and shell structural organization of tag-free Httex1 is influenced by the subcellular environment and the polyQ length but not by the presence of Nt17 domain. The addition of GFP to the C-terminal part of Httex1 induced a differential structural organization. Indeed, no core and shell organization was detected for Httex1 72Q-GFP inclusions, independently of the polyQ length. We demonstrated that neutral lipids are specifically recruited into tag-free Httex1 cellular inclusions in a polyQ length-dependent manner. Moreover, tag-free Httex1 72Q inclusion formation induced mitochondrial fragmentation, increased mitochondrial respiration and led to ER-exit site remodeling. In contrast, Httex1 72Q-GFP inclusion formation did not lead to mitochondrial fragmentation but to reduced mitochondrial respiration and ERES modulation compared to Httex1 72Q. Finally, our quantitative proteomic analysis revealed 55% differences between the co-aggregated proteins with Httex1 72Q compared to Httex1 72Q-GFP. Httex1 72Q inclusions were found to have a specific enrichment of infection and inflammation-related proteins, while Httex1 72Q-GFP exhibited a stronger UPS-related protein enrichment, as well as an enrichment of mitochondria and metabolic-related pathways.

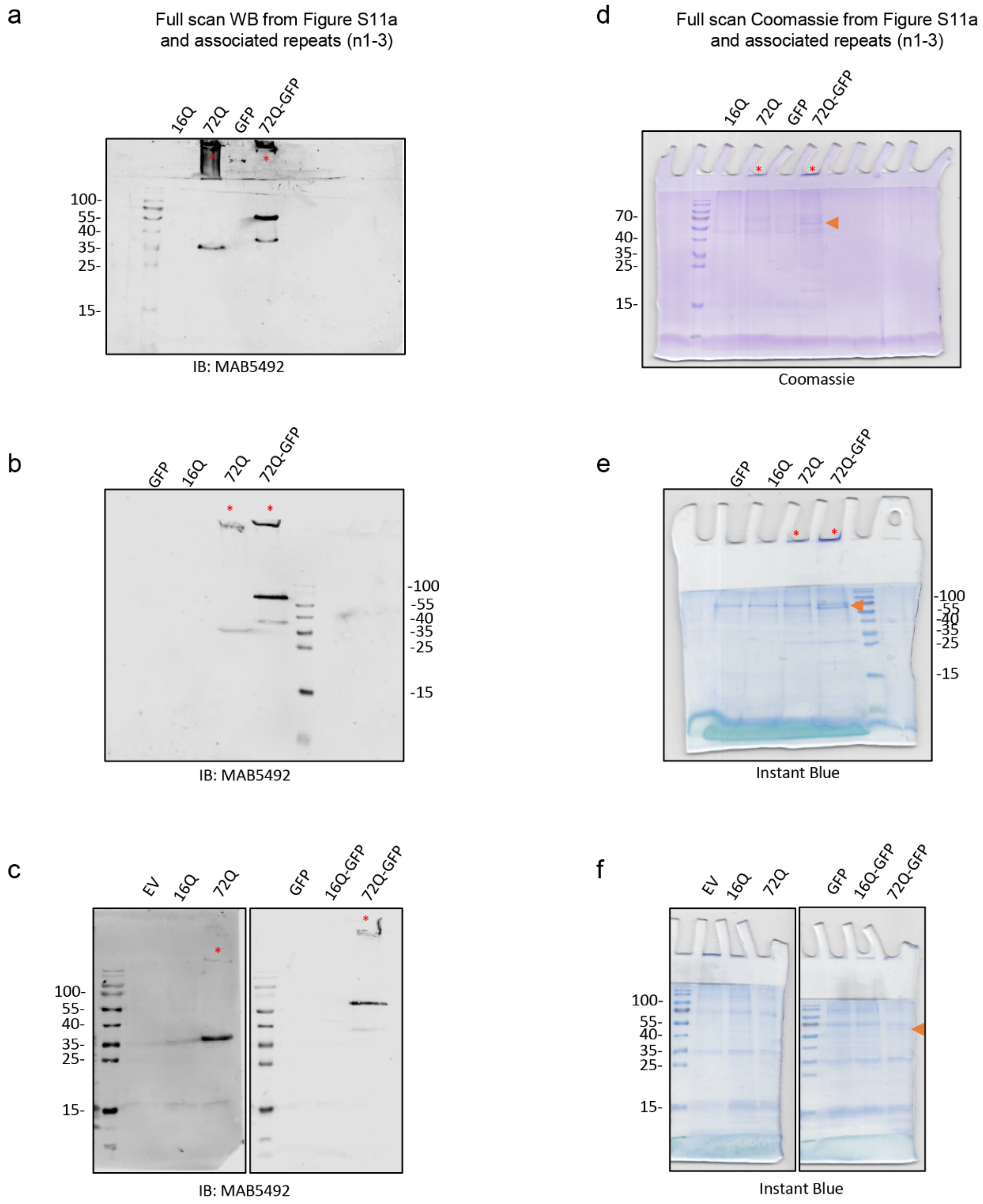

Supplementary Figure 31. **Full scan WB and Coomassie from the Supplementary Figure 11a and associated repeats (n=3). a-c** WB analyses of the Urea fraction revealed only Httex1 72Q and Httex1 72Q-GFP, indicating a good fractionation of the aggregates. **d-f** Coomassie or instant blue detection of total proteins from the Urea fraction. Red stars indicate the presence of aggregates in the stacking gel. The orange arrow indicates the expected size of Httex1 72Q-GFP. Empty vector (EV). Source data are provided as a Source Data file.

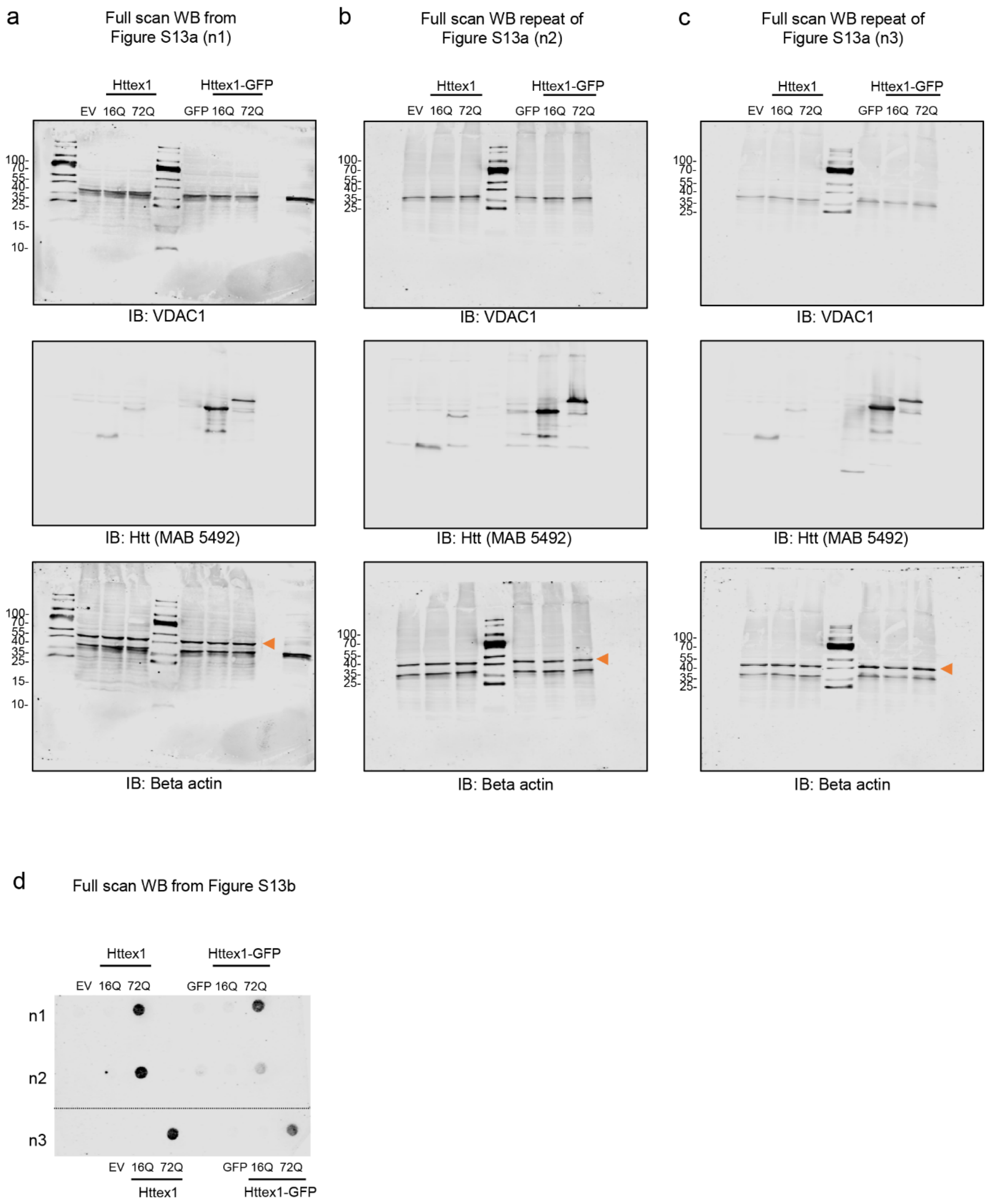

Supplementary Figure 32. **Full scan WB from the Supplementary Figure 13 and associated repeats (n=3). a-c** WB analyses and repeats from the Supplementary Figure 13a with the detection of the outer mitochondrial membrane protein VDAC1, Httex1 and Beta actin (orange arrowhead). **d** Filter trap analyses with repeats on the same membrane from Supplementary Figure 13b with the detection of Httex1 72Q and Httex1 72Q-GFP as large SDS-insoluble aggregates. Empty vector (EV). Source data are provided as a Source Data file.
