# Supplementary material for: Nuclear and cytoplasmic huntingtin inclusions exhibit distinct biochemical composition, interactome and ultrastructural properties
Source: Nat Commun. 2021 Nov 12;12:6579. doi: 10.1038/s41467-021-26684-z (PMC8589980; doi:10.1038/s41467-021-26684-z)
Supplement: Supplementary file 3 — Description of Additional Supplementary Files [file 41467_2021_26684_MOESM3_ESM.pdf]

Description of Additional Supplementary Files

Supplementary Data 1: Comparative analysis of known Htt interactors present in Httex1 inclusions. List and comparison of known Htt interactors present in the proteomic datasets, identified from the HDinHD database (https://www.hdinhd.org/) and restricted to the human and mouse datasets among cell- or animal-based studies exclusively. The columns indicate the known Htt interactors found in HEK cells significantly enriched in Httex1 72Q vs. 16Q (column 1), Httex1 72Q-GFP vs. 16Q (column 2); and in neurons significantly enriched in Httex1 72Q vs. 16Q (column 3) and Httex1 72Q-GFP vs. 16Q (column 4).

Supplementary Movie 1: Httex1 72Q 3D model. 3D reconstruction of Httex1 72Q cytoplasmic inclusion and surrounding organelles formed in HEK cells (48 h post-transfection) based on electron microscopy images. The Httex1 inclusion shell is represented in purple, the core in cyan and intra-inclusion membranous structures are displayed in white. ER membranes are colored in green, the nucleus is in blue, and the mitochondria are shown in yellow. The movie was generated with the Blender® 3D modeling software (Blender Foundation).

Supplementary Movie 2: Httex1 39Q 3D model. 3D reconstruction of Httex1 39Q cytoplasmic inclusion and surrounding organelles formed in HEK cells (48 h post-transfection) based on electron microscopy images. The Httex1 inclusion is represented in purple, and intra-inclusion membranous structures are displayed in white. ER membranes are colored in green, the nucleus is in blue, and the mitochondria are shown in yellow. The movie was generated with the Blender® 3D modeling software (Blender Foundation).

Supplementary Movie 3: Httex1 72Q neuronal tomogram. Tomogram reconstruction of a neuronal Httex1 72Q inclusion seven days post-transduction. White filaments represent the segmented filamentous structures present in neuronal intranuclear inclusions based on the semi-automated tomogram annotation in EMAN2 (see Methods section: "Electron tomography").
