# Supplementary material for: Nuclear and cytoplasmic huntingtin inclusions exhibit distinct biochemical composition, interactome and ultrastructural properties
Source: Nat Commun. 2021 Nov 12;12:6579. doi: 10.1038/s41467-021-26684-z (PMC8589980; doi:10.1038/s41467-021-26684-z)
Supplement: Supplementary file 8 — Reporting summary [file 41467_2021_26684_MOESM8_ESM.pdf]

nature portfolio

Corresponding author(s): Hilal A. Lashuel

Last updated by author(s): Sep 16, 2021

Reporting Summary

Nature Portfolio wishes to improve the reproducibility of the work that we publish. This form provides structure for consistency and transparency in reporting. For further information on Nature Portfolio policies, see our Editorial Policies and the Editorial Policy Checklist.

Statistics

For all statistical analyses, confirm that the following items are present in the figure legend, table legend, main text, or Methods section.

n/a	Confirmed	
☐	☒	The exact sample size (*n*) for each experimental group/condition, given as a discrete number and unit of measurement
☐	☒	A statement on whether measurements were taken from distinct samples or whether the same sample was measured repeatedly
☐	☒	The statistical test(s) used AND whether they are one- or two-sided<br>*Only common tests should be described solely by name; describe more complex techniques in the Methods section.*
☐	☒	A description of all covariates tested
☐	☒	A description of any assumptions or corrections, such as tests of normality and adjustment for multiple comparisons
☐	☒	A full description of the statistical parameters including central tendency (e.g. means) or other basic estimates (e.g. regression coefficient) AND variation (e.g. standard deviation) or associated estimates of uncertainty (e.g. confidence intervals)
☐	☒	For null hypothesis testing, the test statistic (e.g. *F*, *t*, *r*) with confidence intervals, effect sizes, degrees of freedom and *P* value noted<br>*Give P values as exact values whenever suitable.*
☒	☐	For Bayesian analysis, information on the choice of priors and Markov chain Monte Carlo settings
☒	☐	For hierarchical and complex designs, identification of the appropriate level for tests and full reporting of outcomes
☒	☐	Estimates of effect sizes (e.g. Cohen's *d*, Pearson's *r*), indicating how they were calculated

Our web collection on statistics for biologists contains articles on many of the points above.

Software and code

Policy information about availability of computer code

Data collection	For proteomic studies, protein identification and quantification were performed with the search engine MaxQuant 1.6. 2.10 (https://www.maxquant.org/).<br>Zen (https://www.zeiss.fr/microscopie/produits/microscope-software/zen.html) and Leica LasX softwares wereused to collect confocal images (https://www.leica-microsystems.com/fr/produits/logiciel-du-microscope/informations-detaillees/leica-las-x-ls/).<br>The Tomo 4.0 software was used to acquire tilt series of EM samples (https://www.fei.com/software/tomography-4-for-life-sciences/#gsc.tab=0)

Data analysis	FIJI (1.52) and Zen (2.3) were used for image processing and analysis (https://imagej.net/Fiji) and (https://www.zeiss.com/microscopy/int/products/ microscope-software/zen-lite.html). Photoshop CC 2019 was used to align stacks of electron microgrphs prior to segmentation and 3D reconstruction. A custom-developed machine learning-based pipeline, tailored specifically to 3D microscopy data was used from www.ariadne-service.ch. Blender® 3D (v2.79) was used as a modeling software (Blender Foundation: https://www.blender.org/). Inspect 3D v4.1.2 was used for tilt series alignments and tomogram reconstruction (https://www.thermofisher.com/fr/fr/home/electron-microscopy/products/software-em-3d-vis/inspect-3d-software.html). EMAN2 (build after 03/20/2020) was used for filling missing wedge of the resulting tomogram and semi-automated convolutional neural network (CNN) for tomogram annotation (https://blake.bcm.edu/emanwiki/EMAN2). 3dmod from IMOD package (4.11) was used to generate images and movies of the tomogram (https://bio3d.colorado.edu/imod/).<br>Image Studio 3.1 from LiCor (https://www.licor.com/bio/image-studio-lite/) was used for western blotting intensity signal quantification. Perseus software was used for protein identification and filtration (https://maxquant.net/perseus/). DAVID 6.8 (https://david.ncifcrf.gov/home.jsp), Venn diagram website (http://bioinformatics.psb.ugent.be/webtools/Venn/), IPA software (Winter 2019 Release) (https://digitalinsights.qiagen.com/products-overview/ discovery-insights-portfolio/analysis-and-visualization/qiagen-ipa/), R (version 4.0.3) and microsoft Excel (v1808) were used for proteomic analysis. The R code is available in the following repository: https://github.com/jannahastings/httex-proteomics-202107. Graph Pad Prism 9.1.1 was use to generate some of the figures and statistical tests (https://www.graphpad.com/scientific-software/prism/). Kaleidagraph (RRID:SCR_014980) was also used for statistical tests (https://ritme.com/fr/logiciels/kaleidagraph/).

For manuscripts utilizing custom algorithms or software that are central to the research but not yet described in published literature, software must be made available to editors and reviewers. We strongly encourage code deposition in a community repository (e.g. GitHub). See the Nature Portfolio guidelines for submitting code & software for further information.

Data

Policy information about availability of data

All manuscripts must include a data availability statement. This statement should provide the following information, where applicable:

- Accession codes, unique identifiers, or web links for publicly available datasets
- A description of any restrictions on data availability
- For clinical datasets or third party data, please ensure that the statement adheres to our policy

All data in the manuscript or the supplementary information will be made available upon request:
- Raw confocal images of the figures (Fig 1, 2, 4, 5, 7, 8, S2, S5, S6, S9, S10, Sl4, S15, S16, S21, S22 and S29)
- Raw electron microscopy images of the figures (Fig 1, 2, 4, 6, 7, 8,S3, S4, S7, S8, S15, S16, S17, S19, S20, S21 and S29)
All proteomic databases are available via ProteomeXchange (PRIDE) with the identifier PXD021742 for HEK cells and PXD028323 for primary neurons.
The Uniprot databases for Human (Homo Sapiens, release 2019_06) or mouse (Mus musculus, release 2020_10) are available on (https://www.uniprot.org/)

Field-specific reporting

Please select the one below that is the best fit for your research. If you are not sure, read the appropriate sections before making your selection.

☒ Life sciences ☐ Behavioural & social sciences ☐ Ecological, evolutionary & environmental sciences

For a reference copy of the document with all sections, see nature.com/documents/nr-reporting-summary-flat.pdf

Life sciences study design

All studies must disclose on these points even when the disclosure is negative.

Sample size	Sample size were determined based on previous studies using similar methodologies. (https://pubmed.ncbi.nlm.nih.gov/27751235/, https://pubmed.ncbi.nlm.nih.gov/28890085/).
Data exclusions	No data were excluded from the analysis
Replication	Independent experiments were performed to verify the findings (minimum 3 independent experiments) with all attempts at replication successful.
Randomization	This is not relevant to our study as only cellular models were used.
Blinding	Blinding was not relevant to our study as the assays used do not depend from the experimenter.

Reporting for specific materials, systems and methods

We require information from authors about some types of materials, experimental systems and methods used in many studies. Here, indicate whether each material, system or method listed is relevant to your study. If you are not sure if a list item applies to your research, read the appropriate section before selecting a response.

Materials & experimental systems

n/a	Involved in the study
☐	☒ Antibodies
☐	☒ Eukaryotic cell lines
☒	☐ Palaeontology and archaeology
☐	☒ Animals and other organisms
☒	☐ Human research participants
☒	☐ Clinical data
☒	☐ Dual use research of concern

Methods

n/a	Involved in the study
☒	☐ ChIP-seq
☒	☐ Flow cytometry
☒	☐ MRI-based neuroimaging

Antibodies

Antibodies used	2B7 Huntingtin antibody: CHDI, clone 2B7; Ab109115 Huntingtin antibody: Abcam, clone EPR5526, RRID AB_10863082; MWl Huntingtin antibody: CHDI, clone MWl, RRID AB_528290; MAB5492 Huntingtin antibody: Millipore, clone 2B4, RRID AB_11213848; 4C9 Huntingtin antibody: CHDI, clone 4C9; N18 (sc-8767) Huntingtin antibody: Santa-Cruz, clone 3E10, RRID AB_2123254; MW8 Huntingtin antibody: CHDI, clone MW8, RRID AB_528297; S830 Huntingtin antibody: Bates laboratory, s830; EGT414 Huntingtin antibody: Lashuel laboratory, Eurogentec (414- 4D3G9A12) clone 17H01; ab6276 Beta-Actin antibody: Abcam, clone AC-15, RRID AB_2223210; ab21685 BIP/Grp78 antibody: Abcam, RRID AB_2119834; sc-17764 Tom20 antibody: Santa-Cruz, clone F-10, RRID AB_628381; H00008878 p62 antibody: Abnova, clone 2Cll, RRID AB_ 437085; ab92547 Vimentin antibody: Abcam, clone EPR3776, RRID AB_10562134; ab1440 HDAC6 antibody: Abcam, RRID AB_2232905; MAB9055 Sec13 antibody: R&D Systems, clone 1280A; ab14734; VDACl antibody: Abcam, clone 20B12AF2, RRID AB_ 443084; ab92434 MAP2 antibody: Abcam, RRID AB_92434; ab177487 NeuN antibody: Abcam, RRID AB_2532109.
Validation	Antibodies from commercial source have been validated by the vendors and their validation data are available on the manufacturers' website:<br>2B7 Huntingtin antibody: https://www.coriell.org/0/sections/Search/Sample_Detail.aspx?Ref=CH02024&PgId=166, https://pubmed.ncbi.nlm.nih.gov/20086007/; Ab109115 Huntingtin antibody: https://www.abcam.com/huntingtin-antibody-epr5526-ab109115.html, https://pubmed.ncbi.nlm.nih.gov/31941072/, https://pubmed.ncbi.nlm.nih.gov/31844074/; MW1 Huntingtin antibody: https://dshb.biology.uiowa.edu/MW1, https://pubmed.ncbi.nlm.nih.gov/31844074/, https://pubmed.ncbi.nlm.nih.gov/29162692/; MAB5492 Huntingtin antibody: https://www.merckmillipore.com/CH/de/product/Anti-Huntingtin-Antibody-a.a.-1-82,MM_NF-MAB5492, https://pubmed.ncbi.nlm.nih.gov/31088970/, https://pubmed.ncbi.nlm.nih.gov/32070434/; 4C9 Huntingtin antibody: https://www.coriell.org/0/Sections/Search/Sample_Detail.aspx?Ref=CH01272&Product=AB, https://pubmed.ncbi.nlm.nih.gov/29162692/; N18 (sc-8767) Huntingtin antibody: https://www.scbt.com/p/huntingtin-antibody-n-18?requestFrom=search, https://pubmed.ncbi.nlm.nih.gov/21248135/, https://pubmed.ncbi.nlm.nih.gov/21813737/; MW8 Huntingtin antibody: https://dshb.biology.uiowa.edu/MW8, https://pubmed.ncbi.nlm.nih.gov/20086007, https://pubmed.ncbi.nlm.nih.gov/31088970/; ab6276 Beta-Actin antibody: https://www.abcam.com/beta-actin-antibody-ac-15-ab6276.html, https://pubmed.ncbi.nlm.nih.gov/32393636/, https://pubmed.ncbi.nlm.nih.gov/32456010/; ab21685 BIP/Grp78 antibody: https://www.abcam.com/grp78-bip-antibody-ab21685.html, https://pubmed.ncbi.nlm.nih.gov/32409639/, https://pubmed.ncbi.nlm.nih.gov/32393740/; sc-17764 Tom20 antibody: https://www.scbt.com/p/tom20-antibody-f-10, https://pubmed.ncbi.nlm.nih.gov/32034138/, https://pubmed.ncbi.nlm.nih.gov/32364533/; H00008878 p62 antibody: https://www.abnova.com/products/products_detail.asp?catalog_id=H00008878-M01, https://pubmed.ncbi.nlm.nih.gov/32286280/, https://pubmed.ncbi.nlm.nih.gov/32149416/; ab92547 Vimentin antibody: https://www.abcam.com/vimentin-antibody-epr3776-cytoskeleton-marker-ab92547.html, https://pubmed.ncbi.nlm.nih.gov/31512144/, https://pubmed.ncbi.nlm.nih.gov/31830366/; ab1440 HDAC6 antibody: https://www.abcam.com/hdac6-antibody-ab1440.html, https://pubmed.ncbi.nlm.nih.gov/30442948/, https://pubmed.ncbi.nlm.nih.gov/30787326/; MAB9055 Sec13 antibody: https://www.rndsystems.com/products/human-sec13-antibody-1280a_mab9055?utm_source=distributor&utm_medium=referral&utm_campaign=product&utm_term=primaryantibodies; ab14734 VDAC1 antibody: https://www.abcam.com/vdac1-porin-antibody-20b12af2-ab14734.html, https://pubmed.ncbi.nlm.nih.gov/32358564/, https://pubmed.ncbi.nlm.nih.gov/31932578/;<br>ab92434 MAP2 antibody: https://pubmed.ncbi.nlm.nih.gov/32488011/, https://pubmed.ncbi.nlm.nih.gov/31695598/, https://pubmed.ncbi.nlm.nih.gov/31843010/; ab177487 NeuN antibody: https://pubmed.ncbi.nlm.nih.gov/33046105/, https://pubmed.ncbi.nlm.nih.gov/31934864/, https://pubmed.ncbi.nlm.nih.gov/32443895/.<br>Non commercial antibodies were validated by the original lab and against recombinant proteins:<br>S830 Huntingtin antibody:https://pubmed.ncbi.nlm.nih.gov/28465506/, https://pubmed.ncbi.nlm.nih.gov/11689489/; EGT414 Huntingtin antibody was validated against Human Httex1 recombinant proteins and in cells overexpressing Hu

Eukaryotic cell lines

Policy information about cell lines

Cell line source(s)	HEK293 cells: ATCC, [HEK-293T/17] (ATCC® CRL-11268'")
Authentication	HEK293 cells were identified by the vendor: https://www.atcc.org/products/crl-11268 (karyotyping).
Mycoplasma contamination	The HEK293 cells were tested negative to mycoplasma contamination.
Commonly misidentified lines<br>(See ICLAC register)	No commonly misidentified cell lines were used in the study.

Animals and other organisms

Policy information about studies involving animals; ARRIVE guidelines recommended for reporting animal research

Laboratory animals	P0 pups were used from the WT C57BL/6JRj mice strain; Sex: mixed.
Wild animals	No wild animals were used in the study.
Field-collected samples	For primary neuronal culture:<br>Mice pups (P0, mixed sex) are sacrificed by decapitation just before experiments.<br>The mothers will be sacrificed by CO2 inhalation.<br>All experiments will be performed ex vivo.
Ethics oversight	Licence VD3392 established by the Canton de Vaud, Switzerland (Affaires vétérinaires, Ch. des Boveresses 155, 1066 Epalinges)

Note that full information on the approval of the study protocol must also be provided in the manuscript.
